# Structure of Li_3_YCl_6_ and Its Effect on Lithium Ionic Conductivity

**DOI:** 10.1021/jacs.6c06971

**Published:** 2026-09-17

**Authors:** Jue Liu, Yuanpeng Zhang, Zhantao Liu, Paul Cuillier, Paul Cardon, Megan Murphy, Huiwen Ji, Hailong Chen

**Affiliations:** † Neutron Scattering Division, 6146 Oak Ridge National Laboratory, Oak Ridge, Tennessee 37831, United States; ‡ Department of Mechanical Engineering, 1372 Georgia Institute of Technology, Atlanta, Georgia 30332, United States; § Department of Materials Science and Engineering, The Ohio State University, Columbus, Ohio 43210, United States; ∥ Pritzker School of Molecular Engineering, 2462 University of Chicago, Chicago, Illinois 60637, United States; ⊥ Department of Materials Science and Engineering, 43334 The University of Utah, Salt Lake City, Utah 84112, United States

## Abstract

Halide-based Li^+^ solid-state electrolytes have garnered renewed interest in recent years, mainly because of their significantly improved Li^+^ ionic conductivity and excellent stability against high-voltage cathodes. Numerous studies have reported that synthetic conditions and protocols can profoundly influence cation defect chemistry, thereby affecting Li^+^ mobility in these materials. Despite extensive investigations, the structures and fundamental mechanisms governing Li^+^ transport in these compounds remain highly debated, posing challenges for further performance optimization. In this report, we present a comprehensive structural analysis of Li_3_YCl_6_ synthesized under various conditions. Our findings reveal that the structural differences among the various forms of Li_3_YCl_6_ originate from variations in the type and length scales of Y-vacancy ordering within the hexagonal close-packed (*hcp*) anion framework. Our results show that samples treated at lower temperatures tend to exhibit a more uniform distribution of Y^3+^ and vacancies across the two different cation layers. In contrast, high-temperature treatment leads to the preferential occupation of one layer by Y^3+^, while vacancies dominate the other layer. This temperature-driven redistribution underlies the multiple-phase transitions observed upon heat treatment. Kinetic Monte Carlo simulations on the simulated and experimental structures suggest that the Y-vacancy uniformly distributed structure exhibits more balanced in-plane Li^+^ diffusion behavior; in contrast to the Y-vacancy segregated phases, where Li^+^ diffusion is facile in the Y-poor layer but is severely hindered in the adjacent Y-rich layer. This study resolves the longstanding controversies surrounding the structure of Li_3_YCl_6_ and highlights the importance of manipulating the cation-vacancy distribution for optimizing Li^+^ conductivity in *hcp*-based layered halide electrolytes.

## Graphical abstract

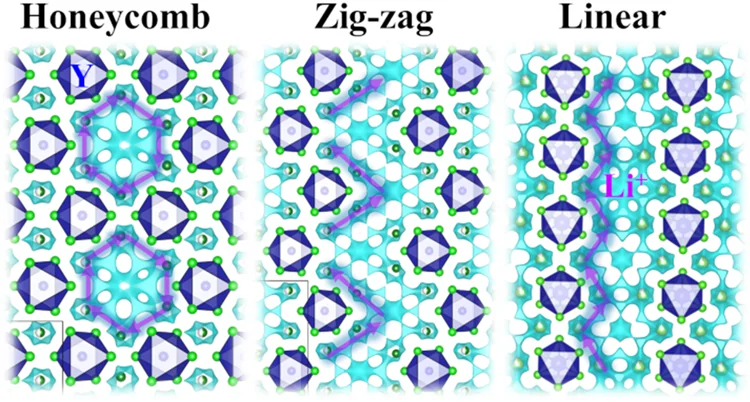

## Introduction

All-solid-state lithium-ion batteries (ASSLIBs) have recently garnered tremendous attention. They offer the potential for higher energy densities and provide superior safety compared to conventional nonaqueous electrolyte-based Li-ion batteries.^1–3^ However, serious hurdles to large-scale commercialization remain, primarily rooted in the significantly reduced interphases between solid electrolytes and electrodes.^2,4–6^ To compensate for this drawback, substantial effort has been devoted to exploring solid-state electrolytes with ultrahigh Li^+^ ionic conductivity at room/low temperatures. This has led to the discovery of various Li^+^ superionic conductors, such as lithium-stuffed garnets,^7,8^ thiophosphates,^9,10^ lithium-rich argyrodites,^11^ and more recently, halide or oxyhalide-based electrolytes.^12–17^ Among these, oxide-based solid-state electrolytes suffer from limited room-temperature Li^+^ conductivity and are often difficult to process due to mechanical stiffness.^2,8^ While sulfide-based electrolytes have the highest Li^+^ conductivity, they present challenges with relatively poor chemical and air stability and are prone to releasing toxic H_2_S gas when exposed to moisture.^18,19^

Layered lithium metal halides with a general formula of Li_3–*x*_MCl_6_ (0 ≤ *x* ≤ 1, M is often 3+ or 4+ rare earth cations or Zr) have recently been revived as promising solid-state electrolyte candidates for ASSLIBs.^12,13,20,21^ Compared to widely used sulfide- or thiophosphate-based electrolytes, halides offer improved interfacial stability against high-voltage cathodes.^20^ However, a major limitation of halide-based electrolytes is their relatively low Li^+^ ionic conductivity. Early reports show that these compounds have relatively low Li^+^ conductivities on the order of 10^–6^–10^–5^ S/cm.^22,23^ Recent advances in synthesis, shifting from conventional solid-state methods to mechanochemical milling, have enabled sub-mS/cm conductivity.^15,21,24^ More recently, through targeted cation and anion substitutions, superionic Li^+^ conductivities exceeding 10 mS/cm have been achieved in newly discovered compositions.^15^ Despite these significant improvements in Li^+^ ionic conductivity, the role of how structure and defects affect Li^+^ transport in layered halide-based electrolytes remains a topic of active debate.^15,25–27^

In layered halide electrolytes with a general formula of Li_3–*x*_MX_6_ (0 ≤ *x* ≤ 1), halide anions form either hexagonal close packing (*hcp*) (Figures S1a–c) or cubic close packing (*ccp*) frameworks (Figure S1d–f), depending on the type of halide and/or the valent/ionic radii of the corresponding cations. Bromides are typically found to adopt the *ccp* anion framework, while the anion stacking sequence for chlorides depends on the ionic radii of rare earth (RE) cations. For instance, Cl^–^ anions follow *ccp* stacking for compounds with small RE cations, such as Li_3_ScCl_6_ or Li_3_InCl_6_. In contrast, compounds with large RE cations, such as Li_3_YCl_6_, Li_3_ErCl_6_, and Li_3_YbCl_6_, have been reported to adopt an *hcp* Cl^–^ framework.^22,23^

In the *ccp* anion frameworks, such as those found in Li_3_YBr_6_ and Li_3_ScCl_6_, the RE cation-containing layers are separated by a cation layer composed solely of Li^+^ ions and vacancies (Figure S1b–S1c). Within the Li^+^ layer, two-thirds of the available octahedral sites are occupied by Li^+^, and the remaining one-third are vacant. In the RE-containing layer, RE cations occupy one-third of the octahedral sites, with Li^+^ occupying the other two-thirds, forming a characteristic RE/Li^+^ honeycomb-ordered pattern (Figure S1c).^24^ These honeycomb-ordered cation layers frequently exhibit stacking faults along the *c*-axis, a structural feature that has been extensively studied in layered compounds such as Li_2_MnO_3_,^28^ Na_3_Ni_2_BiO_6_,^29^ and FeCl_3_.^30^ In our work, as well as in reports from other groups, pronounced stacking faults have been observed in the honeycomb-ordered (vacancy/RE) layers of the Li_3_YBr_6_ (LYB) structure.^24,27,31^ These stacking faults often give rise to asymmetric, sawtooth-like diffuse scattering peaks, commonly referred to as Warren peaks, in powder X-ray or neutron diffraction data.^32^ The impact of stacking faults in these honeycomb-ordered layers on Li^+^ conductivity has attracted increasing interest and has been discussed in several recent studies.^24,26,33^

Although stacking faults have been extensively studied and are generally recognized as the predominant defects in cubic close-packed anion frameworks, such as those in Li_3_YBr_6_ and Li_3_ScCl_6_, the structure and defect chemistry of hexagonal close-packed (*hcp*, ABAB···) anion frameworks remain poorly understood (Figure S1d–f). This has led to ongoing controversies regarding the structural models of these compounds.^25,26,34^ Motivated by these uncertainties, we carried out a detailed investigation of the structure and defect chemistry of Li_3_YCl_6_, a prototypical representative of the anion hcp close-packed layered halide family. This study provides a comprehensive structural overview of various forms of Li_3_YCl_6_ synthesized under different conditions, varying from mechanically milled powders to single-crystal samples. It also sheds light on how to achieve optimized Li^+^ conductivity by fine-tuning the Y/vacancy distribution in these compounds.

## Results and Discussion

### Structure and Defects in Li_3_YCl_6_

In Li_3_YCl_6_ (LYC), the chlorine anions adopt a hexagonal close-packed (*hcp*) structure, following the ABAB··· stacking sequence (Figures 1a–b). The cations occupy the octahedral interstitial sites formed by the chlorine framework, with their positions following a C′C″C′C″··· sequence. In highly crystalline samples synthesized via a solid-state reaction, LYC is widely reported to crystallize in the *P*3̅*m*1 space group (Figure 1a–b). Three distinct Y^3+^ sites are identified in the average structural model: the Y site at the origin (0, 0, 0) is fully occupied, while the other two sites located at *z* = 0.5 and *z* = 0 planes are only partially occupied (Figure 1c–d). The occupancies of these latter sites are highly dependent on the synthetic methods or conditions.^15,21,27^ These partial Y occupancies have been previously attributed to stacking faults in the honeycomb-ordered Y–Li (or vacancy) layers along the stacking direction.^25^ Notably, such stacking faults in the *hcp* framework (ABAB···) result in the formation of face-sharing YCl_6_ octahedra (Figure 2a–b) and form very short Y–Y pairs with an atomic distance of around 3 Å (red arrow in Figure 2b). This configuration is energetically unfavorable due to the strong electrostatic repulsion between closely spaced Y^3+^–Y^3+^ pairs. Furthermore, our neutron and X-ray pair distribution function (PDF) data confirm that Y–Y pairs with ∼3 Å separation are negligible in both the highly crystalline and defect-rich Li_3_YCl_6_ (see Figure S2 for details).^15^ This finding suggests that the apparent partial occupancies refined on the two face-sharing octahedral sites (Y2 and Y3 in the *P*3̅*m*1 structure, Figure 1b) are more likely due to other types of structural defects rather than stacking faults in the honeycomb-ordered cation layers. There are two plausible Y-vacancy defect models in the *hcp* close-packed anions without introducing the aforementioned short Y–Y pairs: one is by forming Y–Li antisite defects, and the other by forming different Y-vacancy arrangements on the C′ (*z* = 0) and C″ (*z* = 0.5) planes. Density functional theory (DFT) calculations (Figure S3) suggest that these arrangements are more energetically favorable than defects that create face-sharing YCl_6_ octahedra.

**Figure 1:**
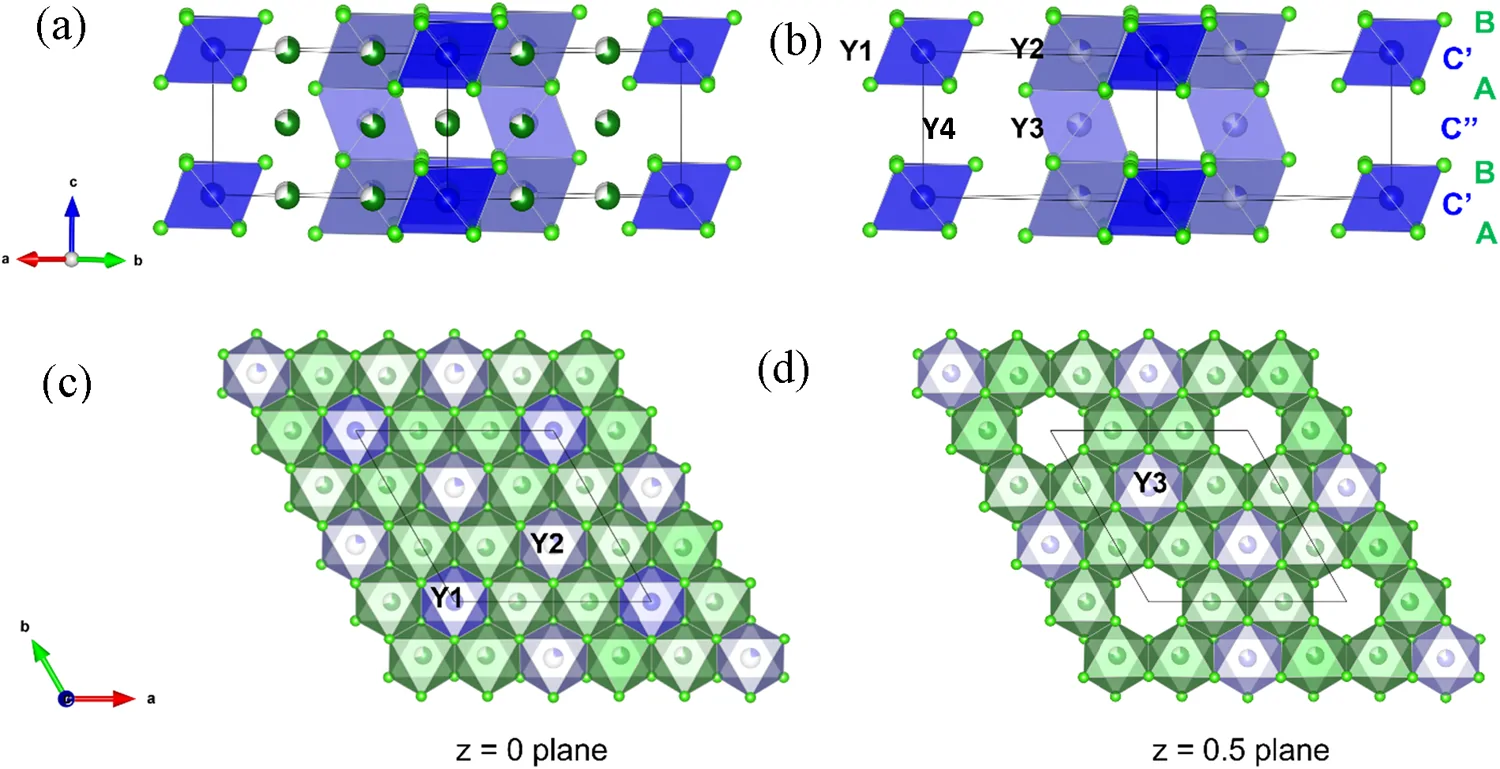
(a) Broadly reported structure of highly crystalline Li_3_YCl_6_ in the space group *P*3̅*m*1. Li, Y, and Cl are shown in olive, light green, and blue spheres. Only YCl_6_ is shown in octahedra for clarity. (b) Chlorine anions adopt the *hcp* close packing with a registration sequence of ABAB···, and there are three distinct Y sites in the structure, while the available vacant octahedral site (Y_4_) below/above the Y1 site is reported to be empty. (c, d) Li and Y occupy 2/3 of the available octahedral sites, leaving the other 1/3 octahedral sites empty. In the fully ordered scenario, Y1 (0,0,0) and Y3 (1/3,2/3,0.5) sites are fully occupied, while Y2 (1/3,2/3,0) site is fully empty. Thus, Y and vacancy form honeycomb-ordered patterns on both *z* = 0 and *z* = 0.5 planes, and the ratios between Y and vacancies are 1:2 and 2:1, respectively. The Y2 and Y3 sites are found to be partially occupied, and the reported average occupancies vary drastically under different synthetic conditions.

**Figure 2:**
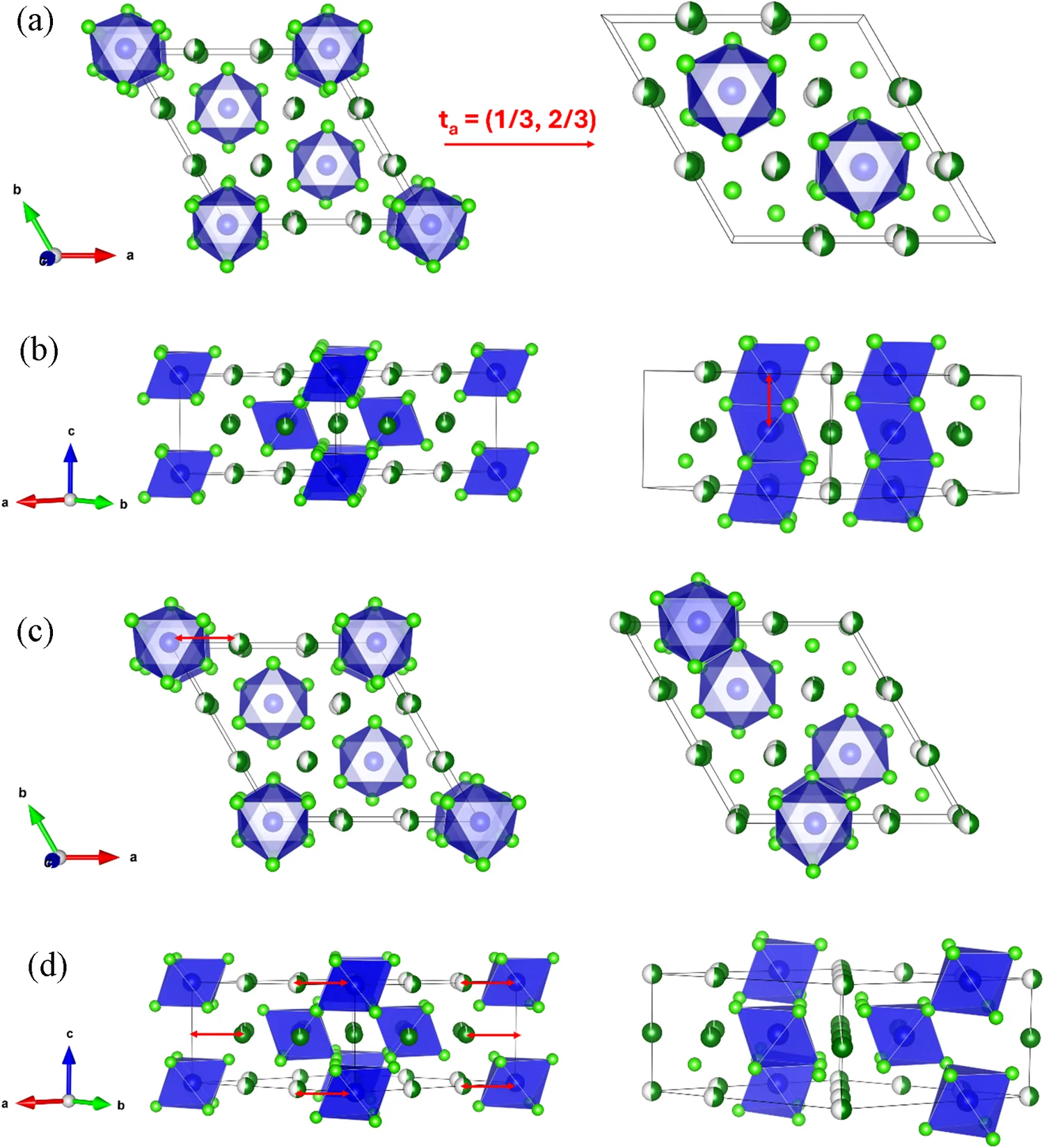
Demonstration of different types of defects in Li_3_YCl_6_. (a, b) Stacking faults of honeycomb-ordered Y-vacancy layers along the *c*-axis direction. The introduction of this type of stacking faults leads to the formation of face-shared YCl_6_ octahedra with a short Y–Y pair distance of 3 Å (highlighted by a red arrow in (b)). (c, d) Demonstration of the formation of Li–Y antisite defects (highlighted by red arrows). Conner- or edge-shared YCl_6_ octahedra are introduced by forming the Li–Y antisite defects. Note: Some lithium atoms are omitted for clarity.

There are two ways to create Y–Li antisite defects in the ideal Y-vacancy honeycomb-ordered structure, one by switching Y on the Y1 site with the neighboring Li/vacancy site (Figure 2c), creating the corner shared YCl_6_ octahedra across the C′ and C″ planes. In this configuration, the shortest Y–Y pair distance is around 4.7 Å (Figure 2c). The other is by swapping Y with the Li site around either Y2 or Y3 site (Figure 2d), leading to the edge-shared YCl_6_ octahedra with the shortest Y–Y pair distance of about 3.8 Å. From the average structure perspective, both antisite defect scenarios would result in either an increase in the average electron density at the Li sites (using XRD) or a decrease in the negative scattering length density (using NPD). Therefore, average structure refinements using either X-ray or neutron powder diffraction should be capable of quantifying the presence of Y–Li antisite defects. We performed Rietveld refinements on samples immediately after ball milling (expected to have the highest defect concentration) and on the highly crystalline sample (annealed at 400 °C and then quenched to room temperature). Refinement results revealed that the concentration of Li–Y antisite defects was very limited for both scenarios (Figures S4–S6, Tables S1–S3), indicating that Y–Li antisite disorder is unlikely to be the dominant defect in LYC.

Thus, the formation of different Y-vacancy arrangements on the C′ and C″ planes (and their length scales) is the most likely defect. In Li_3_YCl_6_, the Li^+^ and Y^3+^ cations occupy two-thirds of the available octahedral interstitial sites formed by the *hcp* close-packed Cl^–^ anion framework. These octahedral sites are evenly distributed between the C′ (*z* = 0) and C″ (*z* = 0.5) planes. As a result, many different configurations are possible for distributing Y^3+^ and Li^+^ among the available octahedral sites, with the constraint that the face-sharing octahedral sites directly above and below the YCl_6_ octahedra must remain vacant to avoid the formation of very short Li–Y or Y–Y pairs (∼3 Å apart). The widely reported honeycomb ordering pattern (Figure 1) represents one such possible ordering arrangement. Figures 3 and S7 illustrate several plausible Y-vacancy ordering patterns on the C′ (*z* = 0) and C″ (*z* = 0.5) planes, ranging from the two most commonly observed ordering arrangements, the honeycomb ordering (Figure 3a) and the zigzag ordering (Figure 3b), to other possible configurations, including linear ordering (Figure 3c), herringbone ordering (Figure 3d), three-line ordering (Figure S7a), mixed zigzag/linear ordering (Figure S7b), and cross-square ordering (Figure S7c). Two configurations representing Y and vacancies randomly distributed on both C′ (*z* = 0) and C″ (*z* = 0.5) planes are also shown in Figure S7e,d. DFT calculations predict negligible energy differences between the honeycomb ordering and linear (−1.7 meV/atom), three-line (0.8 meV/atom), and zigzag (−0.7 meV/atom) ordering patterns, suggesting that it is relatively easy to form mixed domains with different Y-vacancy ordering patterns. This also indicates that it is possible to synthesize Li_3_YCl_6_ with different Y-vacancy ordering patterns by fine-tuning the synthetic conditions (e.g., annealing temperature).

**Figure 3:**
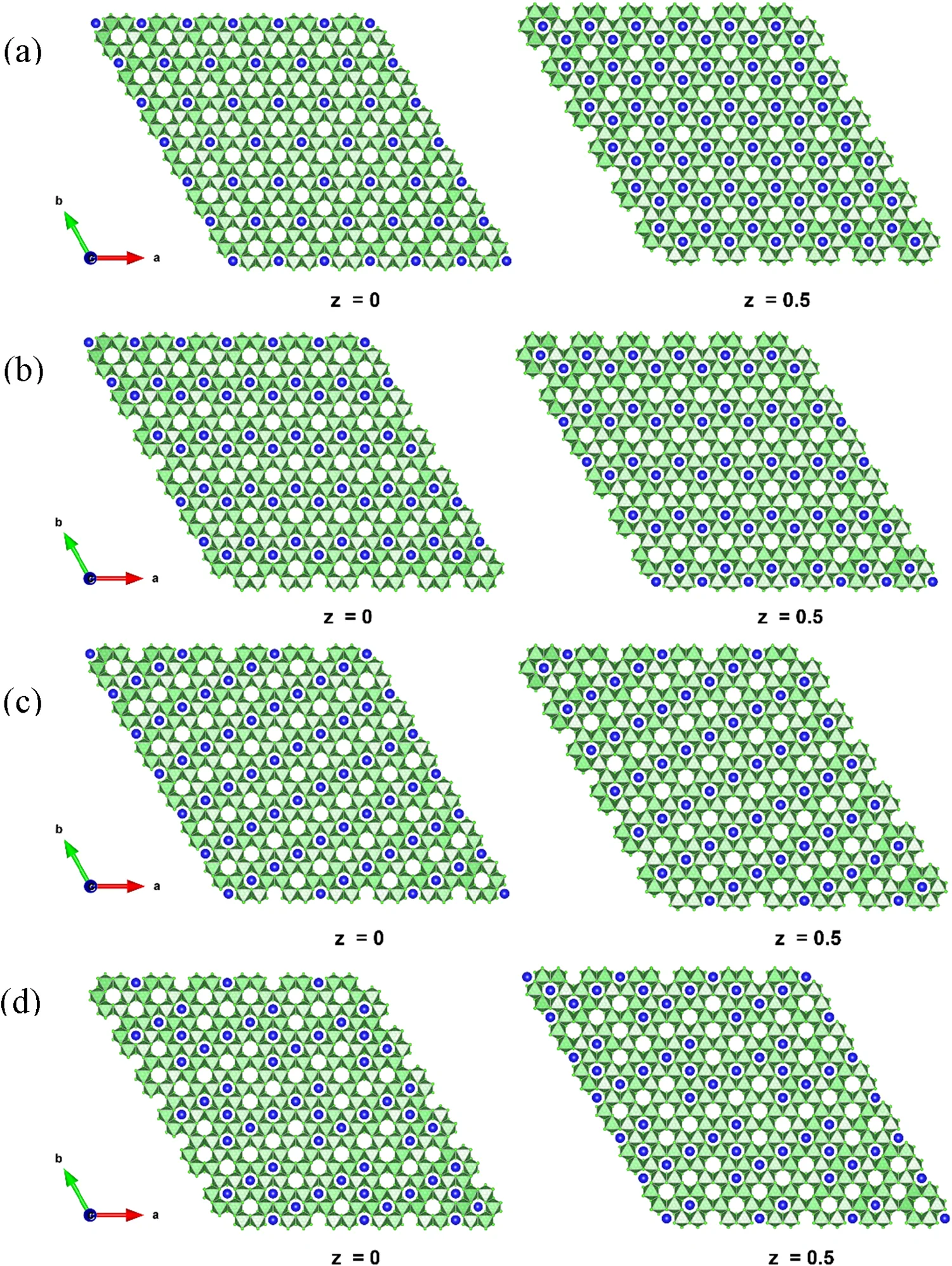
Different Y-vacancy ordering patterns: (a) honeycomb ordering, (b) zigzag ordering, (c) linear ordering, and (**d**) herringbone ordering. The left side shows the cation and vacancy distribution on the C′ plane (*z* = 0), and the right side shows the C″ plane (*z* = 0.5). It is assumed that the 1/4 vacancies on the Li^+^ sites are randomly distributed. The blue spheres represent Y^3+^, the green-colored octahedra represent LiCl_6_, and the vacant octahedral sites are left empty. In the honeycomb-ordered Y-vacancy structure, Y cations and vacancies form a honeycomb ordering pattern on both the C′ and C″ planes, but with opposite Y-to-vacancy ratios. Specifically, the Y:vacancy ratio is 1:2 on the C′ plane and 2:1 on the C″ plane. In contrast, the zigzag, linear, and herringbone ordering motifs all exhibit an equal distribution of Y cations and vacancies on the C′ and C″ planes, with a Y:vacancy ratio of 1:1 on each plane. The distinction among these three ordering motifs lies in the in-plane arrangement of the Y cations and vacancies. In zigzag ordering, the Y cations occupy alternating octahedral sites, forming a characteristic zigzag pattern. In the linear ordering, Y cations occupy contiguous rows of octahedral sites, separated by adjacent rows of vacant octahedral sites. The herringbone ordering can be regarded as an intermediate configuration between the linear and zigzag motifs. Other types of Y-vacancy arrangements can be found in Supporting Information Figure S7.

To gain more insight into the plausible structures with different Y-vacancy arrangements, we simulated the XRD and neutron diffraction patterns (NPD) (Figure 4) from different Y-vacancy ordering patterns (Figures 3 and S7). The intensities of several major reflections are nearly identical across all the different Y-vacancy ordering configurations, indicating that these reflections are primarily governed by the *hcp* stacking of the Cl^–^ anions. In contrast, the superlattice reflections, particularly those in the 3.3–5.2 Å range (highlighted in olive), vary significantly with the specific types of Y-vacancy arrangement. Notably, all superlattice peaks are absent in the model with a fully randomly distributed Y-vacancy (black curve), where all available octahedral sites are half-occupied by Y^3+^. This simulation suggests that the superlattice peaks in the XRD data are highly sensitive to the Y-vacancy arrangements. It is worth noting that the relative intensities of these superlattice peaks are much weaker in the simulated NPD (Figure 4b) due to the negative coherent scattering length of Li.

**Figure 4:**
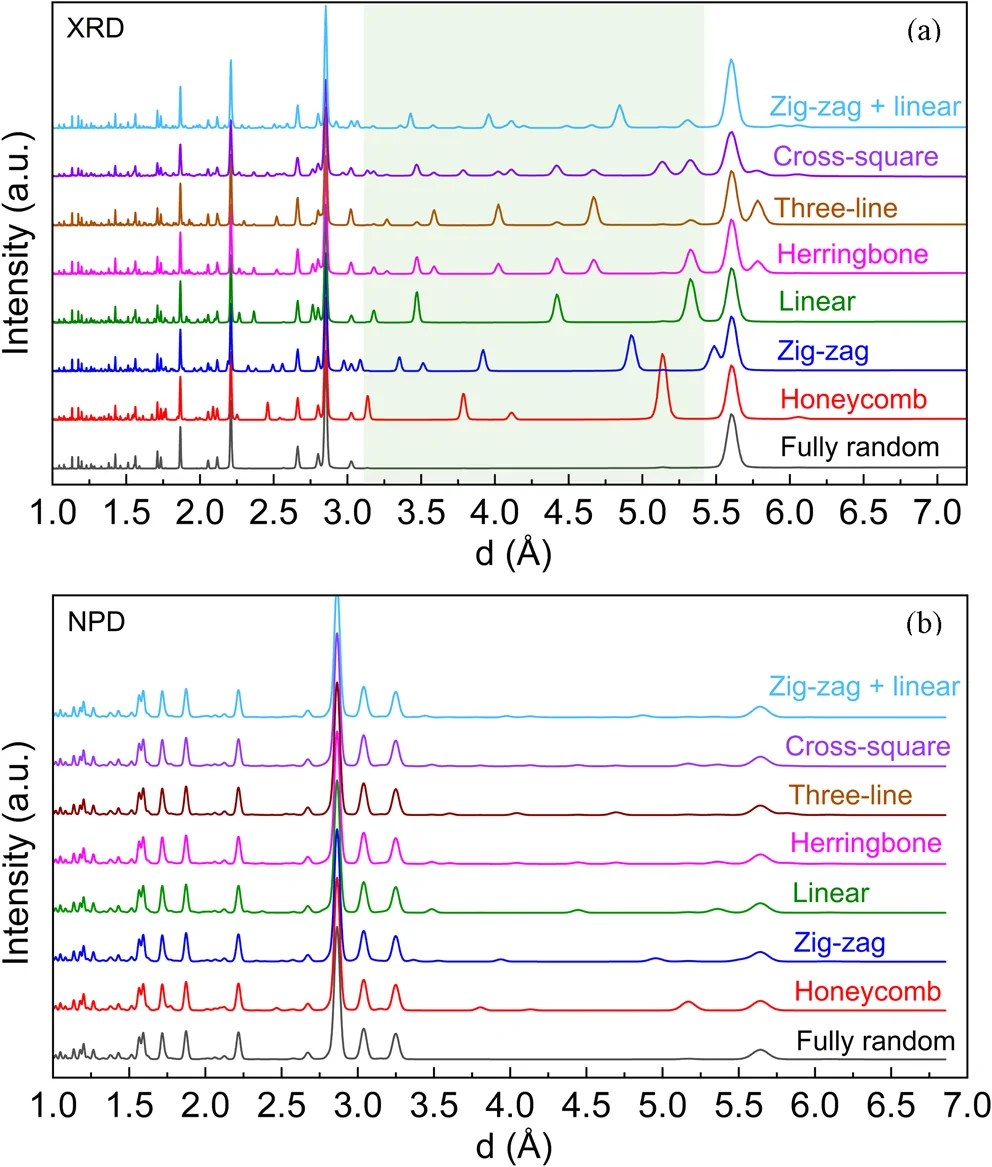
Simulated XRD (a) and neutron diffraction (b) patterns of Li_3_YCl_6_ with different types of Y-vacancy arrangements on the C′ and C″ planes. Note: Instrumental broadening profiles similar to those for the experimental data are convoluted into the simulated data.

These simulations motivated us to carry out in situ XRD to monitor phase transitions in Li_3_YCl_6_ during the heat treatment of the mechanically milled starting material (Figure 5). Sharp reflections associated with the *hcp* close-packed chlorine framework have formed just after ball milling (Figure 5, Phase-1). The starting room-temperature X-ray diffraction pattern (sample just after ball milling) can be reasonably modeled using the *P*3̅*m*1 space group with a ABAB··· stacked chlorine anion framework (Figure S8). Notably, the superlattice peaks are very broad and diffuse, suggesting that the length scale of the Y-vacancy ordering is very limited. As the temperature increases, these diffuse superlattice peaks become sharper until the temperature reaches about 160 °C. Upon further heating, a new set of diffraction peaks emerges at the expense of the original diffuse peaks (see the dotted blue line in Figure 5, Phase-2). This phase transition is completed at about 200 °C. The newly formed phase is maintained between 200 and 260 °C before transforming to a highly crystalline phase above 260 °C (Phase-3). We noticed that the simulated XRD pattern for the ideal honeycomb-ordered Y-vacancy ordering pattern (Figure 4a) matches well with the *in situ* XRD data collected above 260 °C (Figure 5). The superlattice peak positions for the phase formed between 180 and 240 °C match those simulated from the zigzag Y-vacancy-ordered pattern (Figure S9), despite the much broader superlattice peaks in the measured diffraction data. These observations suggest that inter- and/or intraplane migration of Y^3+^/vacancies occurred during the heating of the ball-milled starting material. For instance, the conversion from a honeycomb-ordered Y-vacancy configuration to a zigzag-ordered configuration requires the out-of-plane migration of Y^3+^; the conversion between zigzag-ordered pattern and linear-ordered pattern can be achieved with only in-plane migration.

**Figure 5:**
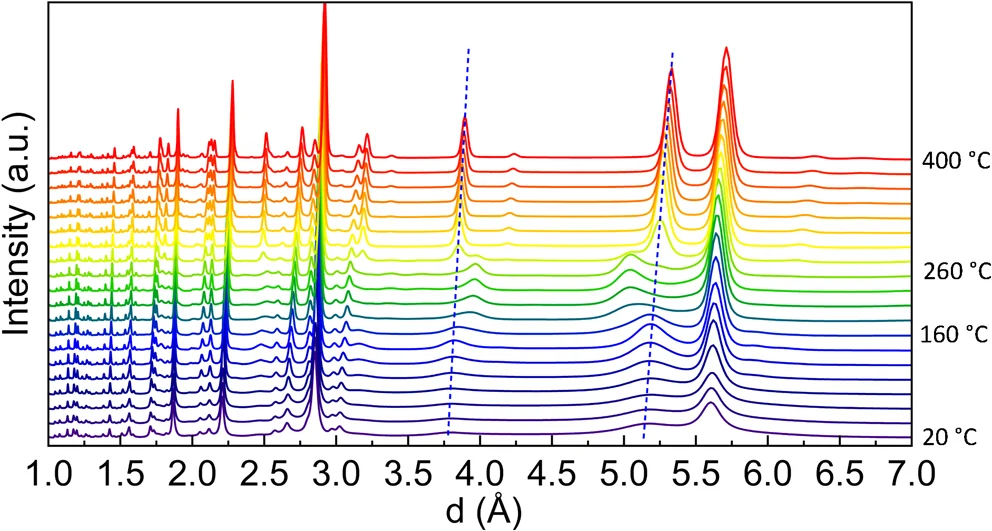
In situ XRD patterns (λ = 0.24117 Å) collected during heating of the ball-milled starting materials from 20 to 400 °C with a step size of 20 °C/step. There are three different phases formed during the heating process: first, from 20 to 140 °C, the second from 160 to 240 °C, and the third above 260 °C. The dotted blue lines along the superlattice peaks highlight the transformation of three different phases. We label the corresponding three regions as the initial ball-milled phase 1 (Phase-1), intermediate-ordered phase 2 (Phase-2), and long-range ordered phase 3 (Phase-3).

Moreover, the sharp superlattice peaks (associated with the Y-vacancy honeycomb ordering) broaden moderately after cooling below 320 °C (Figure S10), suggesting that Y^3+^ also migrates during the cooling process. Thus, a quench is required to obtain highly crystalline Li_3_YCl_6_ with near-ideal Y-vacancy honeycomb ordering. It is also worth noting that small amounts of a new impurity phase emerge at temperatures above 320 °C, together with a slight increase of the LiCl impurity. This new phase shows much smaller thermal expansion relative to the main Li_3_YCl_6_ phase, as evidenced by the almost unchanged diffraction peak positions during heating to 400 °C (highlighted in Figure S11). Since the *hcp* close-packed anion framework remains almost unchanged during the entire heating process, the various phase transformations are very likely caused by changes in the type and length scales of Y-vacancy ordering in the structure.

Rietveld refinements were then carried out to refine the average structure for samples just after ball milling, and samples heated to 140 °C, 220 and 320 °C (Figures S12–13 and Tables S5, S8–S9). Y-vacancy honeycomb-ordered model (S.G. *P*3̅*m*1) was used to fit the XRD data for the samples just after ball milling (collected at 20 °C) and those heated to 140 °C, 220 and 320 °C. Four different Y sites are introduced during the refinement: the Y1 (0, 0, 0), Y2 [(1/3,2/3,0) or (2/3,1/3,0)], and Y3 [(1/3, 2/3,0.5) or (2/3,1/3,0.5)] sites are the three commonly reported Y sites, and the Y4 site (0,0,0.5) is a face-shared octahedral site with the Y1 site (Figure 1b). The Y4 site is introduced to capture the average distribution of Y and vacancies on C′ and C″ planes (Y2 and Y3 sites, Y1 and Y4 sites can be viewed as paired face-shared YCl_6_ sites on the C′ and C″ planes; only one site can be occupied in the real structure). We found that the positions of the superlattice peaks calculated from the S.G. *P*3̅*m*1 model match well with the experimental XRD data for the samples just after ball milling and those heated to 140 °C and 320 °C (Figures S12a, S12b, and S12d). However, the positions of the calculated superlattice peaks are very different from those in the experimental XRD data for the sample heated to 220 °C (Figure S12c), particularly for the two strongest superlattice peaks at *d*-spacings of around 3.8 Å and 5 Å (highlighted in Figure S12c). We found that the strongest superlattice peaks in the 220 °C heated sample can be better indexed with the Y-vacancy zigzag-ordered model (S.G. *Pnma*, Figure S13). Despite the reasonable match of the diffraction peak positions, the superlattice peaks are much broader and more diffuse in most samples (e.g., the ball-milled and heated at 140 and 220 °C), except for the sample heated at 320 °C (Figure S12), suggesting that the coherent lengths are limited for specific Y-vacancy ordering schemes in the low-temperature-treated Li_3_YCl_6_ samples. This has been further confirmed by Rietveld refinements against neutron diffraction data collected at 300 and 500 K (Figures S14–S15 and Tables S4, S6–S7)

To further elucidate the short- to medium-range Y-vacancy ordering in the low-temperature-treated phases, it is necessary to develop a structural refinement method capable of reconstructing plausible short-range ordering patterns from the corresponding diffraction data. This method must not only effectively exclude configurations containing face-sharing YCl_6_ octahedra, which are energetically unfavorable (Figure S3), but also reconstruct the correct type and length scales of Y-vacancy ordering from a Y-vacancy randomly distributed initial model. To evaluate whether the Y-vacancy ordering pattern can be effectively reconstructed from a randomly distributed Y-vacancy model, we generated a 12*a* × 12*b* × 1*c* supercell (comprising 4320 atoms) based on a Rietveld-refined average structure with a space group of *P*3̅*m*1. In this supercell, Li^+^ ions, along with 1/6 of the vacant octahedral sites, occupy only the original LiCl_6_ octahedral positions, without mixing with the Y sites; that is, no Li–Y antisite defects are introduced (see Figure 2 and Figure S3 for more details). Y^3+^ was then randomly distributed on the available octahedral sites (note: the ratio between Y^3+^ and the available interstitial octahedral sites is 1:2) by using a random generator. A key constraint was applied during supercell generation: if a Y^3+^ ion occupies a specific site at (*x*_1_, *y*_1_, 0) on the C′ layer, the corresponding face-sharing site at (*x*_1_, *y*_1_, 0.5) on the C″ layer is left vacant, and vice versa. This setup effectively eliminates face-shared Y^3+^–Y^3+^ pairs. Therefore, the two extreme cases are (i) Y^3+^ only resides on either the *z* = 0 or *z* = 0.5 plane, which is essentially the M33 model in Figure S3b; and (ii) Y^3+^ is equally distributed among the two layers, such as the Y-vacancy linear, zigzag, or herringbone ordered models shown in Figure 3b–d.

A few examples of randomly generated supercell configurations that satisfy this constraint are shown in Figure S7d,e. These models were then refined against the simulated XRD patterns (with various Y-vacancy ordering patterns, Figure 3) using the nonlinear least-squares refinement method. Polynomial backgrounds and the occupancy of Y^3+^ on the available octahedral sites were allowed to be refined during the reconstruction process. A distance penalty function of the nearest Y–Y pair was introduced to drive away the formation of 3 Å Y–Y pairs during refinement (detailed penalty function can be found in Supporting Information sections S1 and S2). The reconstruction of several different Y-vacancy ordering patterns from randomly distributed initial models was tested, including Y-vacancy honeycomb, zigzag, linear, herringbone, three-line, cross-square, and mixed zigzag and linear ordering. For the honeycomb, zigzag, linearly, three-line, and herringbone ordering tests, we found that the ordering patterns can be effectively reconstructed after the convergence of the initial Rietveld refinement cycle with very different random starting Y-vacancy configurations (Figures 6 and S16). However, the reconstruction of the cross-square and mixed zigzag and linear ordering patterns failed after several attempts using different randomly distributed Y-vacancy initial models (see Figure S17 for more details). A close inspection shows that the superlattice peaks from the simulated XRD pattern of these two ordering patterns are much weaker and span a large range of *d*-spacings (Figure 4a). This might lead to the nonlinear least-squares refinements being stuck at the local minimum (Figure S17).

**Figure 6:**
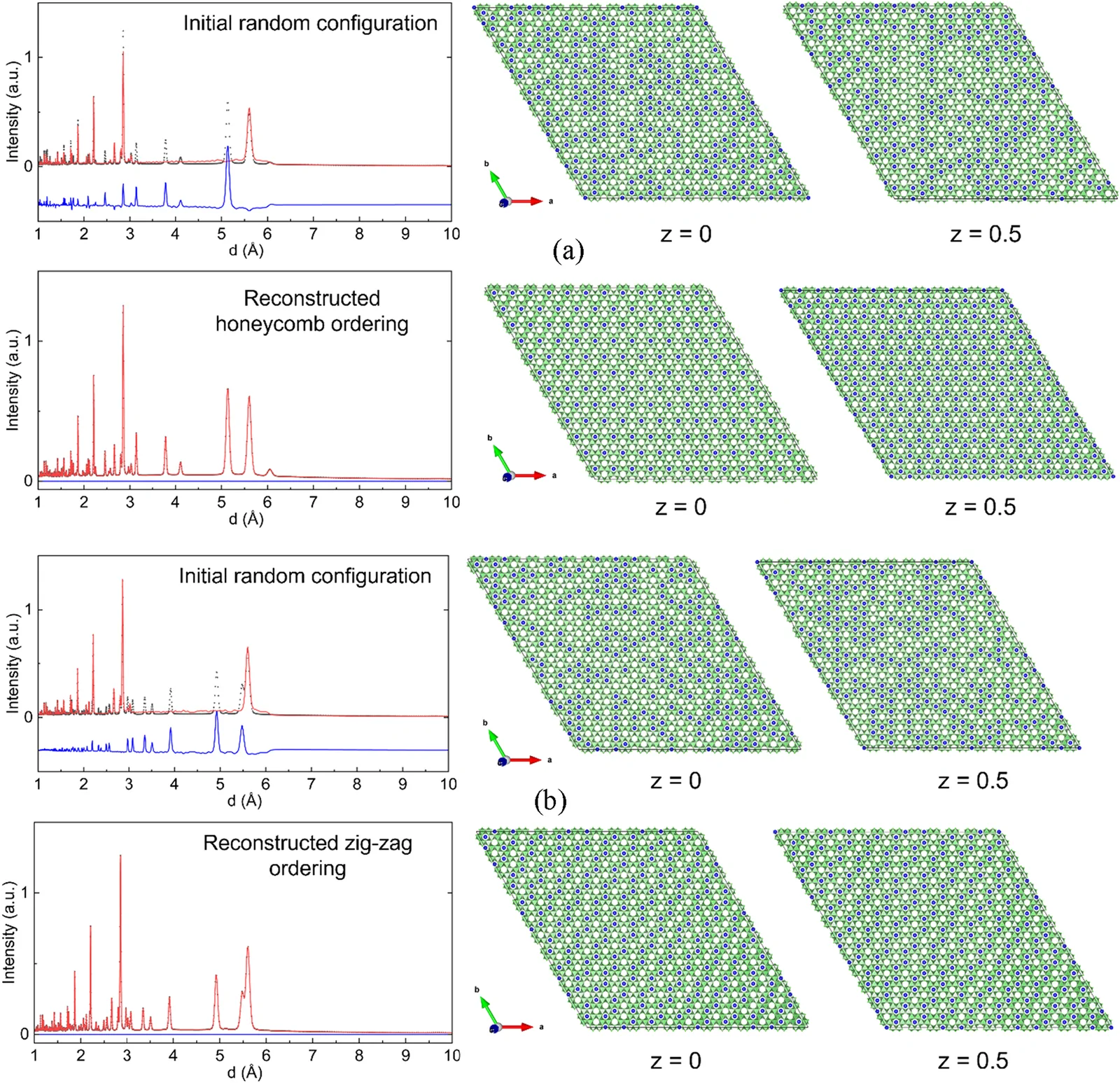
Reconstructing the Y-vacancy ordering patterns from the Y-vacancy randomly distributed initial model (either fully occupied or fully empty Y site) using nonlinear least-squares structure refinements against simulated XRD patterns. Reconstruction of (a) honeycomb ordering and (b) zigzag ordering. Y^3+^ is shown as blue spheres, olive octahedra-colored represent LiCl_6_, and vacant octahedral sites are left empty. The upper left of each figure shows the fit of the XRD data (calculated curve in red, simulated data in black, and difference curve in blue) using the initial random model (upper right). The bottom left shows the converged fit with the successfully reconstructed Y-vacancy ordered model (bottom right).

To enable the refinement to search for the global minimum, we tested two methods: a simple random-walk Monte Carlo (MC) method and a simulated annealing (SA) method. For the random-walk Monte Carlo method, a new refinement cycle is restarted after the convergence of the previous Rietveld refinement cycle using a newly generated randomly distributed Y-vacancy model. More than 100 refinement cycles (a refinement cycle is defined as being converged once the χ^2^ change is smaller than 0.001) were carried out so that enough sampling space of initial configurations can be explored (details can be found in Supporting Information S1). We found that the combined random-walk MC and Rietveld refinement can reconstruct the Y-vacancy cross-square ordering pattern, typically after several attempts of randomization at the initial Y-vacancy arrangements (Figures 7a and S18a). However, the reconstruction of mixed zigzag and linear ordering often failed to emerge even after tens of refinement cycles (Figure S18b). This motivated us to develop a combined simulated annealing (SA) and nonlinear least-squares refinement method to achieve a more effective search for the global minimum.^35,36^ For this method, we first started a nonlinear least-squares refinement cycle to obtain the local minimum configuration. Then, a temperature regime is defined so that the initial Y^3+^ occupancies for the subsequent refinement cycles can be changed according to the defined temperature sequences (more details can be found in Supporting Information S2), mimicking the melting (high temperature) and annealing (low temperature with a slight decrease of temperature) processes. We found that this method can very effectively reconstruct the mixed zigzag/linear ordering and other types of Y-vacancy ordering patterns (Figures 7b and S19). The results above underscore that the position, shape, and intensity of superlattice peaks in the XRD data are highly sensitive to the specific type of Y-vacancy ordering in Li_3_YCl_6_. This motivated us to investigate the plausible configurations and length scales of Y-vacancy orderings using in situ XRD data collected during the heating of ball-milled Li_3_YCl_6_.

**Figure 7:**
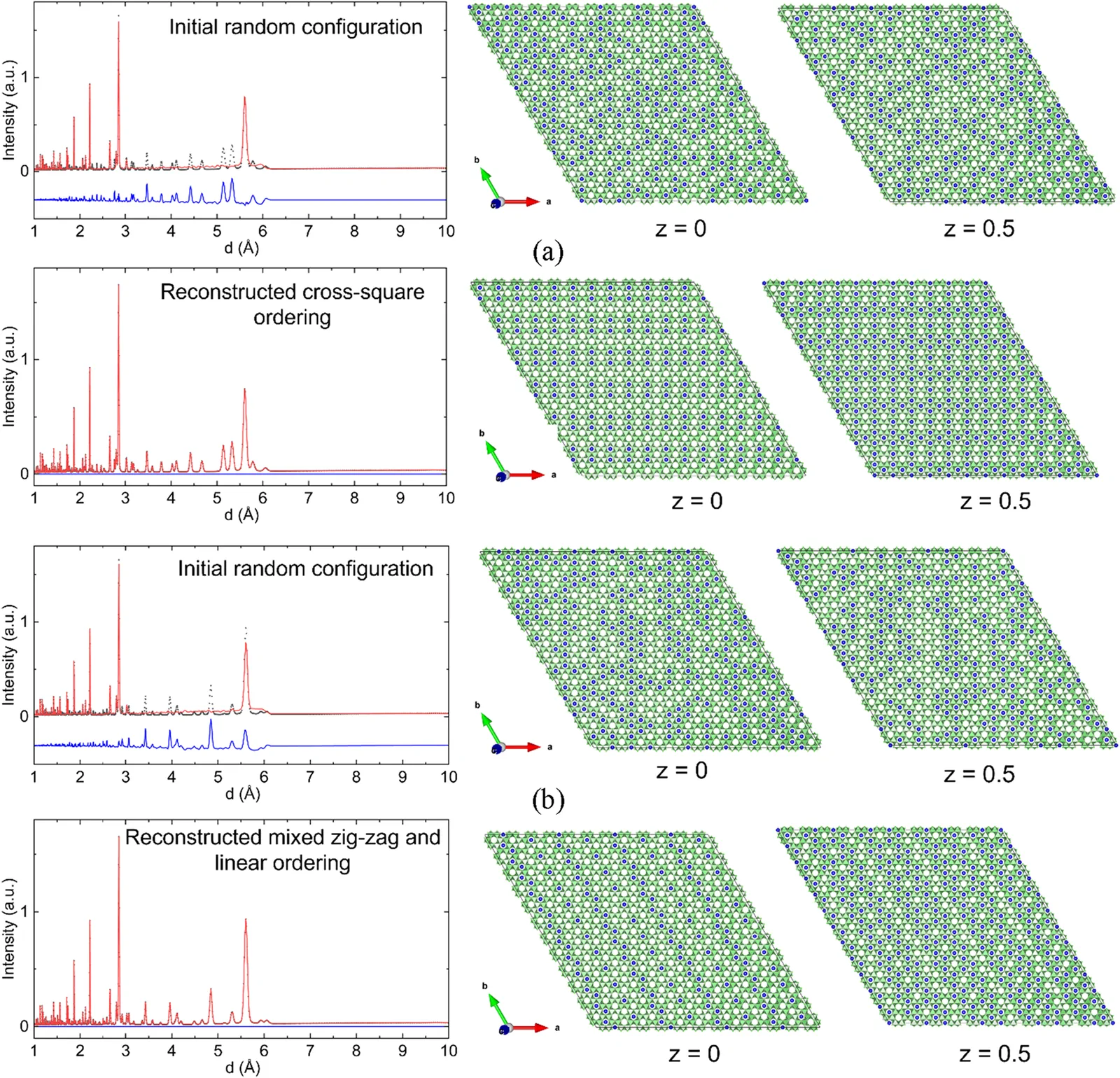
(a) Reconstruction of the cross-square Y-vacancy ordering model from the Y-vacancy randomly distributed model using combined Monte Carlo and nonlinear least-squares refinements. (b) Reconstruction of the mixed zigzag and linear Y-vacancy ordering model from the Y-vacancy randomly distributed model using combined simulated annealing and nonlinear least-squares refinement. Y^3+^ is shown as blue spheres, olive-colored octahedra represent LiCl_6_, and vacant octahedral sites are left empty. The upper left of each figure shows the fit of the XRD data (calculated curve in red, simulated data in black, and difference curve in blue) using the initial random model (upper right). The bottom left shows the converged fit with the successfully reconstructed Y-vacancy ordered model (bottom right).

We first studied the sample immediately after ball milling. Notably, the hexagonal close-packed (*hcp*) Cl^–^ anion framework (ABAB···) is already established at this stage, as indicated by the presence of relatively sharp reflections associated with anion stacking, specifically the (2̅10) and (30̅1) reflections, within the average structure (S.G. *P*3̅*m*1) (Figures 5 and S8). In contrast, the superlattice peaks associated with the Y-vacancy ordering are broadened drastically, a phenomenon that has been previously attributed to the stacking disorder of Y-vacancy-ordered honeycomb layers along the *c*-axis (or the stacking direction).^25^ However, we found that there are no observable 3 Å Y–Y pairs in either the X-ray or the neutron PDF data, and the energy required to form these short Y–Y pairs is relatively high (Figure S3), suggesting that there is a very limited amount of stacking faults in the honeycomb-ordered Y-vacancy layers. It is also worth noting that the superlattice peaks are symmetrically broadened, in clear contrast to the typical sawtooth-shaped stacking disordering peaks of honeycomb-ordered cation layers. Thus, the broadening of these superlattice peaks is very likely caused by the limited coherent lengths of the different in-plane Y-vacancy ordering domains.

To explore the types and length scales of Y-vacancy ordering in this sample, we carried out large-box modeling (the 12*a* × 12*b* × 1*c* supercell expanded from the Rietveld-refined structure) using the combined SA and nonlinear least-squares refinement against the experimental XRD data (Figure 8). Good agreement between the calculated results and experimental data has been achieved after a few cycles of converged refinements. In the refined structure, Y^3+^ ions and vacancies are not randomly distributed but instead form clusters with relatively short coherence lengths on both the C′ and C″ layers (Figure 8b). The ratio of the amounts of Y on C′ and those on the C″ layer is refined to around 1.2, which is much lower than that in the ideal Y-vacancy honeycomb-ordered model (where this ratio should be 2, see Figure 3a). A close inspection revealed that within a given layer, Y^3+^ ions tend to concentrate in specific regions, forming a quasi-honeycomb ordering between the YCl_6_ and LiCl_6_ octahedra (highlighted in Figure 8b). In contrast, other regions contain more sparsely distributed Y cations, giving rise to either Y-vacancy or vacancy–Li honeycomb-ordered configurations. These findings highlight that the broad diffuse scattering signals observed in the powder XRD data are caused by the very limited coherence of the Y-vacancy ordering domains on the C′ (*z* = 0) and C″ (*z* = 0.5) planes.

**Figure 8:**
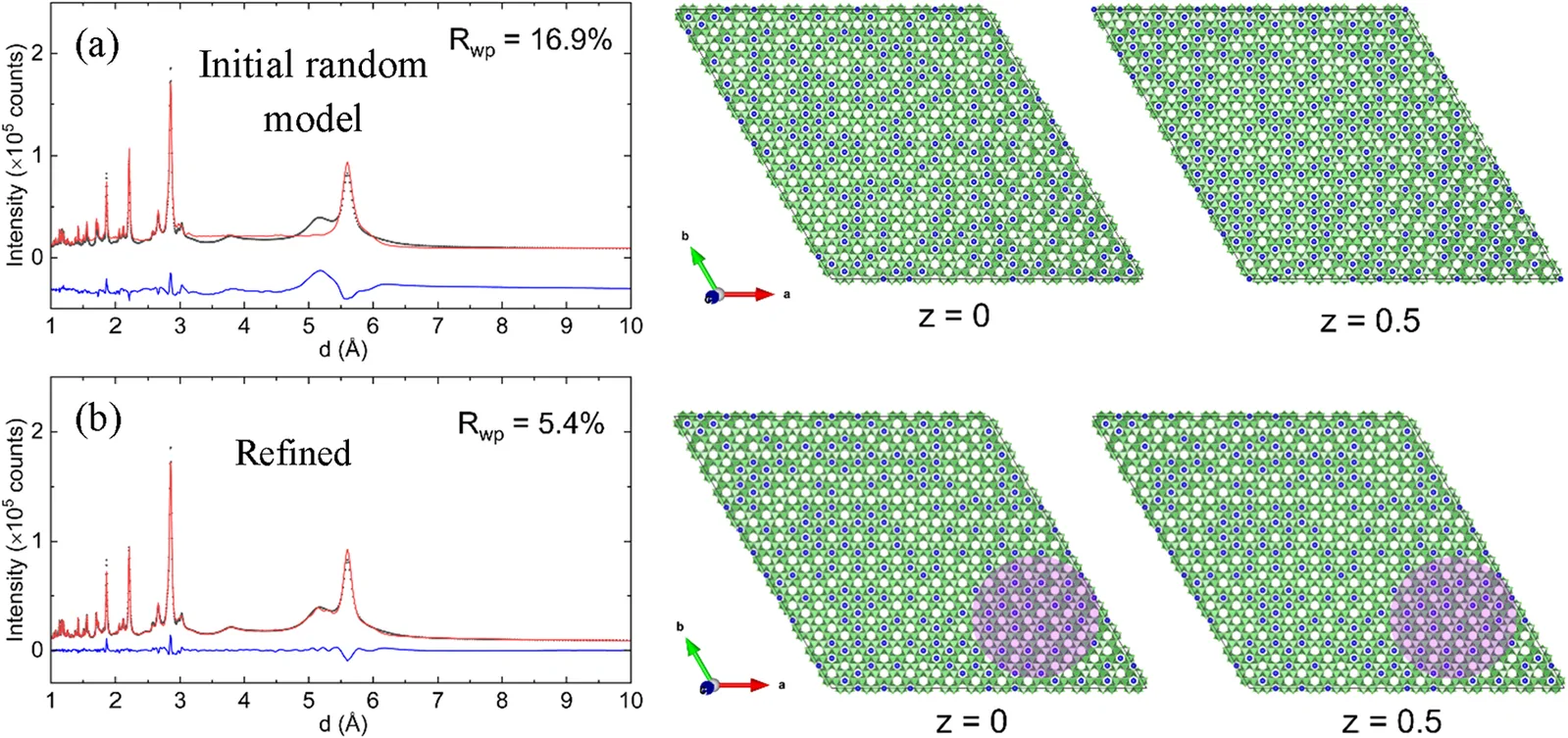
(a) Left: fit of the XRD data of Li_3_YCl_6_ just after ball milling with the randomly distributed Y-vacancy model. Right: (ab-plane) projection of the *z* = 0 and *z* = 0.5 planes of the model. The LiCl_6_ octahedra are shown in olive, Y is shown as blue spheres, and vacant sites are left empty. (b) Left: fit result of the XRD data after simulated annealing and convergence of the least-squares refinements. Right: structure refined from the combined SA and least-squares refinement.

To further confirm the validity of the refined Y-vacancy distribution model, corefinement was carried out using equally weighted XRD, neutron Bragg diffraction, and neutron pair distribution function (nPDF) data. For the initial testing of the corefinement, Y and vacancy distributions are fixed to the values obtained from the refinement using XRD alone (Figure 8b), and the atomic coordinates are also fixed. The lattice parameters and atomic displacements are allowed to be refined separately for different data sets. Reasonable fits can be achieved for XRD, neutron diffraction, and long-range neutron PDF data (12–25 Å). However, there are noticeable discrepancies between the calculated results and the short-range neutron PDF data (1.5–5 Å), particularly around the two shortest chemical bonds (Li–Cl and Y–Cl bonds, highlighted in Figure S20). This suggests that the distributions of the Li–Cl and Y–Cl bonds are much broader than those refined from the long-range diffraction data. To further capture this broad distribution of halide-related bonds, the atomic coordinates of Cl^–^ were allowed to be refined with the Li–Cl and Y–Cl bond length constraints (by limiting the degree of changes in the atomic coordinates, i.e., Δ*x*, Δ*y*, and Δ*z*). The final refinement results are shown in Figure 9. These results further confirm that the superlattice peaks in the XRD data can be used to effectively reconstruct the Y-vacancy ordering clusters in Li_3_YCl_6_, despite the fact that the short-range coordination and bond lengths may vary from those obtained from average structural refinements.

**Figure 9:**
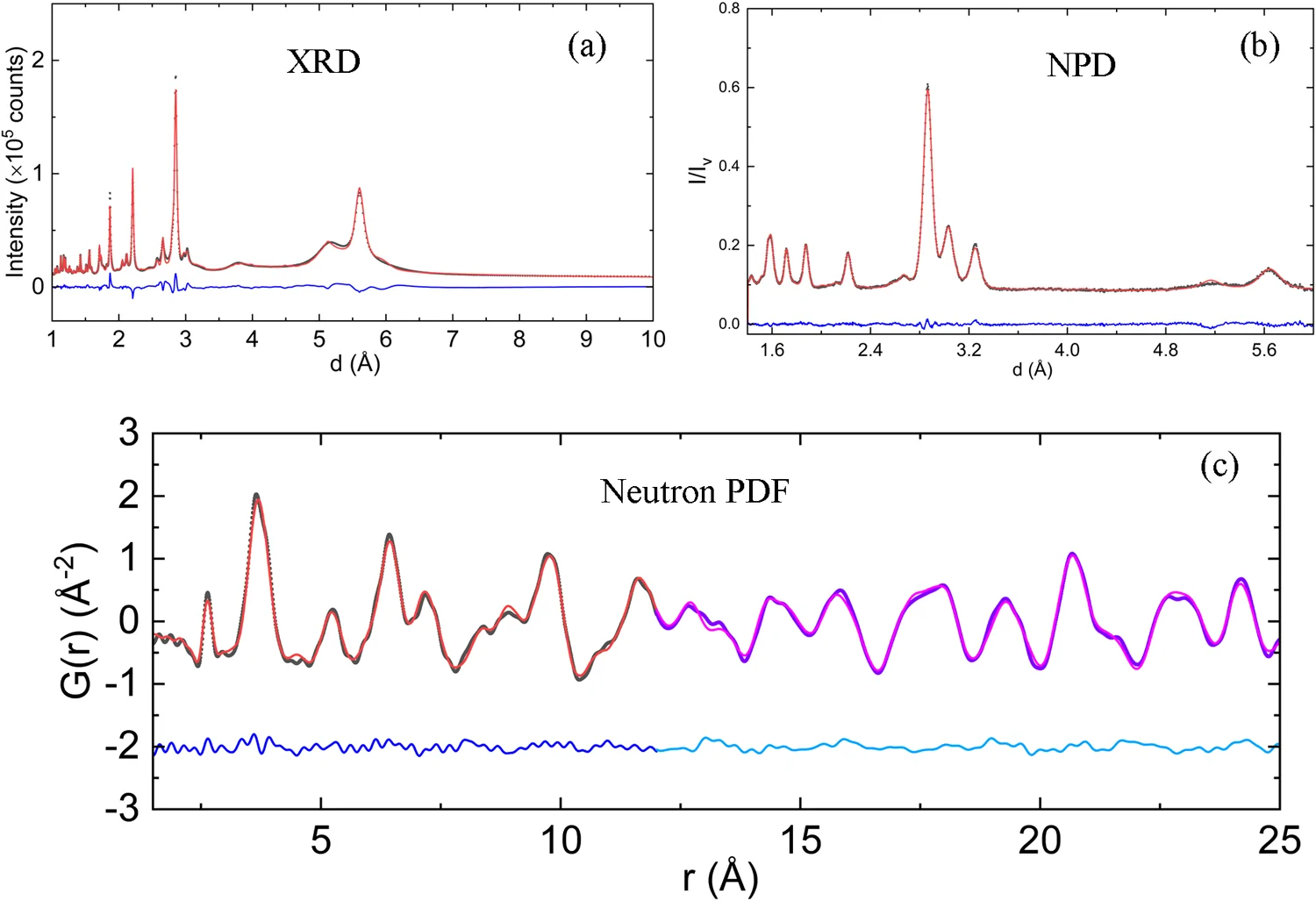
Corefinement of the large-box model using equally weighted XRD (a), neutron diffraction (b), and neutron pair distribution function data (c). The calculated result for the short-range neutron PDF data is shown as a red curve, while the calculated result for the long-range neutron PDF data is shown in pink.

As the temperature increases, the superlattice peaks associated with Y-vacancy ordering become sharper (Figure 5), though they remain broader than the primary diffraction peaks associated with anion stacking. Rietveld refinement using the Y-vacancy honeycomb-ordered model (*P*3̅*m*1) yields a significantly improved fit compared to the refinement of the ball-milled sample (Figure S12b). The Rietveld refinement results show that the occupancy ratio of Y^3+^ ions on the *z* = 0 (C′) and *z* = 0.5 (C″) planes is approximately 1.7, which is slightly lower than the ideal value of 2 expected for perfect Y-vacancy honeycomb ordering (Figure 3a). However, the superlattice peaks are still much broader than the simulated ideal honeycomb ordering data (Figure S12b), suggesting that the coherent lengths of the Y-vacancy honeycomb ordering domains are limited. This is indeed consistent with our large-box modeling (Figure 10), where an excellent agreement between the experimental data (including diffuse superlattice peaks) and calculated results has been achieved. The refined structure shows large regions of Y-vacancy ordering (ratio 2:1) in the *z* = 0 plane, while there are large regions of vacancy-Y honeycomb ordering (ratio 2:1) in the *z* = 0.5 plane. There are also noticeable regions with Y-vacancy zigzag and linear ordering (Figure 10b).

**Figure 10:**
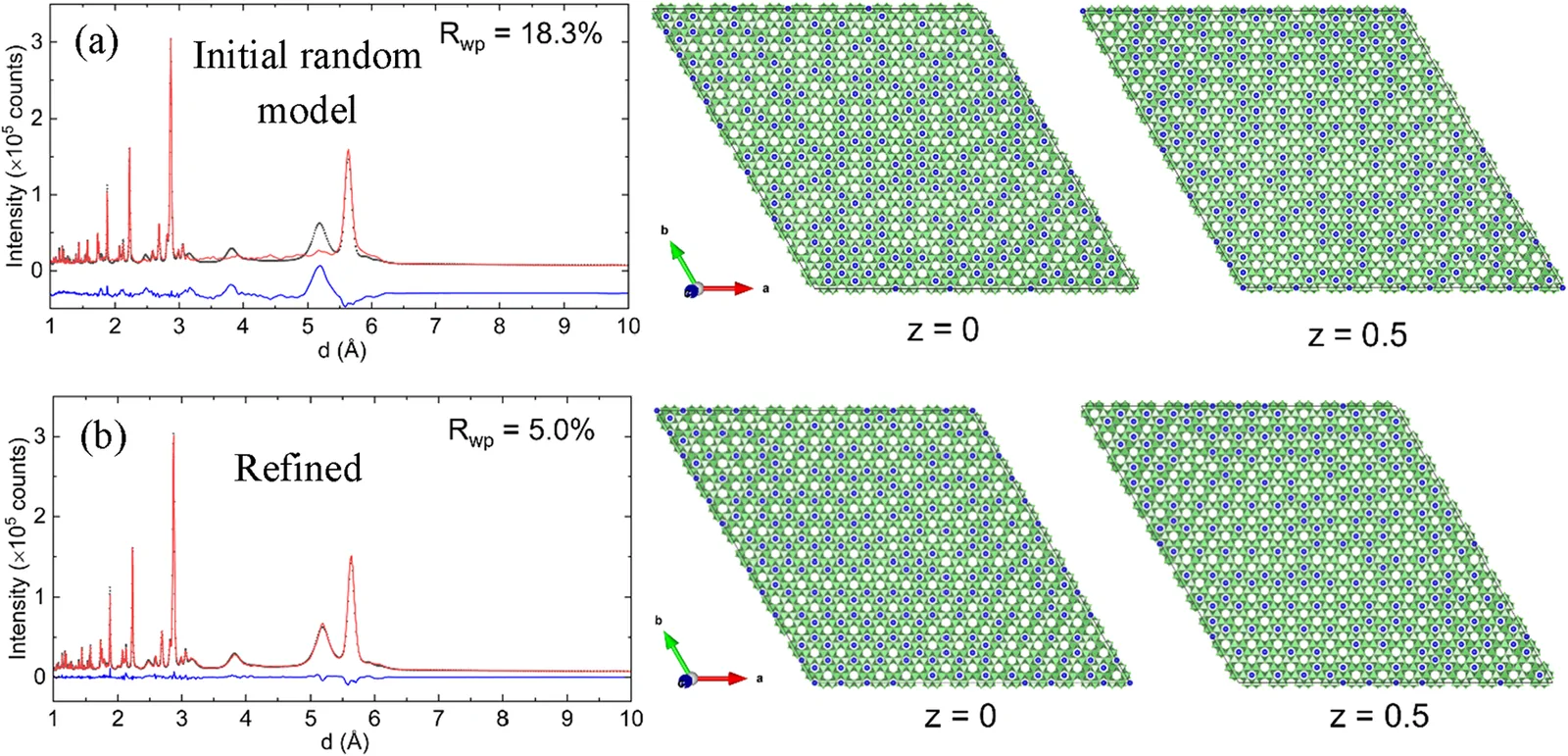
(a) Left: fit of the XRD data of Li_3_YCl_6_ heated to 140 °C with a randomly distributed Y-vacancy model. Right: (ab-plane) projection of the *z* = 0 and *z* = 0.5 planes of the model. The LiCl_6_ octahedra are shown in olive, Y is shown as blue spheres, and vacant sites are left empty. (b) Left: fit result of the XRD data after simulated annealing and convergence of the least-squares refinements. Right: structure refined from the combined SA and least-squares refinement.

When the sample is further heated to above 140 °C, a new set of superlattice peaks emerges, coinciding with the disappearance of the original superlattice reflections (see the blue dotted line in Figure 5). Specifically, the peak near 5.5 Å shifts to smaller *d*-spacings, while the peak around 3.8 Å shifts to larger *d*-spacings. This structural transition is completed at approximately 220 °C, beyond which the superlattice peaks begin to gradually shift back in the opposite direction. The newly formed superlattice reflections can only be poorly fitted using the Y-vacancy honeycomb-ordered model (Figure S12c), suggesting the emergence of a distinct Y-vacancy ordering pattern at elevated temperatures. Indeed, the relatively sharp portion of the superlattice peaks can be reasonably modeled using the Y-vacancy zigzag-ordered model (S.G. *Pnma*), despite the poor fit of diffuse scattering intensities (Figure S13). These observations suggest that a significant portion of the Y^3+^ ions and vacancies adopt a zigzag ordering pattern, coexisting with other types of short-range ordering schemes. Large-box structure refinement, based on a random initial distribution of Y and vacancies (generated from the refined average structure model in S.G. *Pnma*), successfully reconstructs a plausible Y-vacancy ordering pattern, while also providing a satisfactory fit to both the Bragg peaks and diffuse scattering features in the XRD data (Figure 11). The refined structure indeed confirms that Y-vacancy ordering is dominated by the zigzag-ordered pattern, despite the noticeable amounts of Y-vacancy honeycomb-ordered regions.

**Figure 11:**
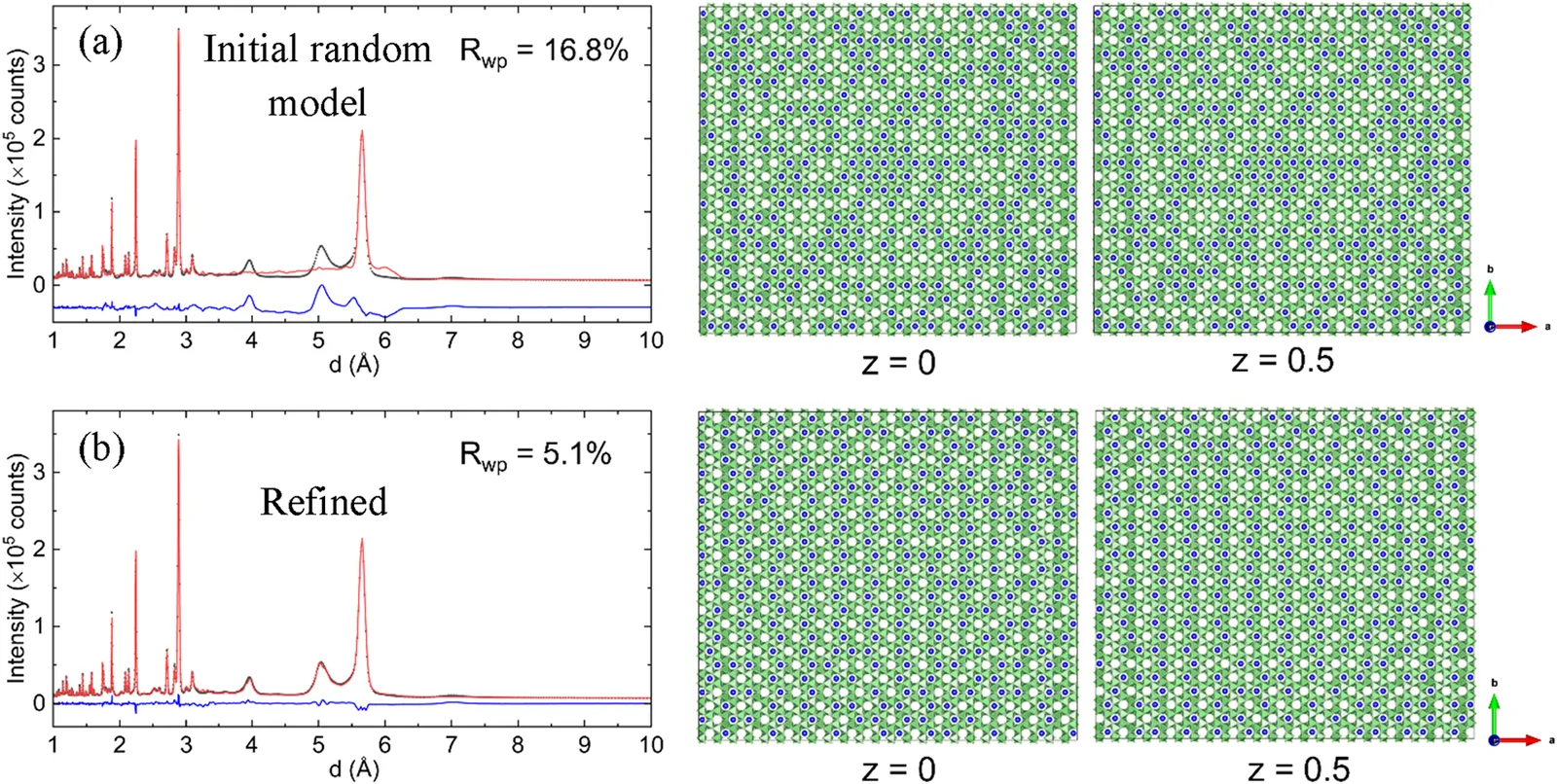
(a) Left: fit of the XRD data of Li_3_YCl_6_ heated to 220 °C with a randomly distributed Y-vacancy model. Right: (ab-plane) projection of the *z* = 0 and *z* = 0.5 planes of the model. The LiCl_6_ octahedra are shown in olive, Y is shown as blue spheres, and vacant sites are left empty. (b) Left: fit result of the XRD data after simulated annealing and convergence of the least-squares refinements. Right: structure refined from the combined SA and least-squares refinement.

Another structural transition occurs when the sample is heated above 240 °C, leading to the formation of a highly crystalline phase (Figure 5). Unfortunately, a minor impurity phase appeared when the sample was heated above 340 °C (Figure S11). Therefore, we focus our structural analysis on the sample annealed at 320 °C, which exhibits a minimal amount of this impurity phase. The diffraction pattern of this highly crystalline phase can be reasonably modeled using the Y-vacancy honeycomb-ordered average structure in the *P*3̅*m*1 space group (Figure S12d). In this refined model, the average cross-plane Y-site mixing on the Y2 sites (Figure 1b) is approximately 12%, suggesting that there is a small degree of Y-vacancy disorder embedded within the long-range honeycomb-ordered lattice. To further probe the level of disordering in the structure, we performed large-box model refinements (Figure S21), and the statistical analysis of the Y^3+^ distribution on the two planes (*z* = 0 and *z* = 0.5) yields a ratio of around 1.42. This closely matches the site-mixing ratio (∼1.45) between the neighboring face-sharing YCl_6_ octahedral sites (M2 and M3 sites in Figure 1), as determined by Rietveld refinement (Figure S12d and Table S10). From the refined structure, we found that the major defects are a mixing of the two Y-vacancy honeycomb ordering patterns on the same plane, i.e., some regions with a Y:vacancy ratio of 2:1, while the other regions with a ratio of 1:2, leading to a lower average Y ratio between the *z* = 0 and *z* = 0.5 planes (Figure S21).

We noticed that the superlattice peaks associated with Y-vacancy ordering broaden during cooling (Figures S10). The broadening of these superlattice peaks suggests a decrease of the coherent lengths of the Y-vacancy honeycomb ordering domains. This is very likely caused by Y^3+^/vacancy migration during the cooling process. Rietveld refinement against the XRD data for the sample that is cooled down to 40 °C (from 400 °C) suggests that the average cross-plane Y-site mixing on the Y2 sites (Figure 1b) has increased to about 17% (Table S12). This is also consistent with our large-box modeling (Figure 12), where the statistical analysis of the Y^3+^ distribution on the two different z planes (*z* = 0 and *z* = 0.5) shows a ratio of around 1.35 (184 Y atoms on the *z* = 0 plane and 248 Y atoms on the *z* = 0.5 plane). This result suggests that quenching is needed to preserve very high degrees of Y-vacancy honeycomb ordering, a standard practice that has been broadly adopted to synthesize highly crystalline Li_3_YCl_6_.

**Figure 12:**
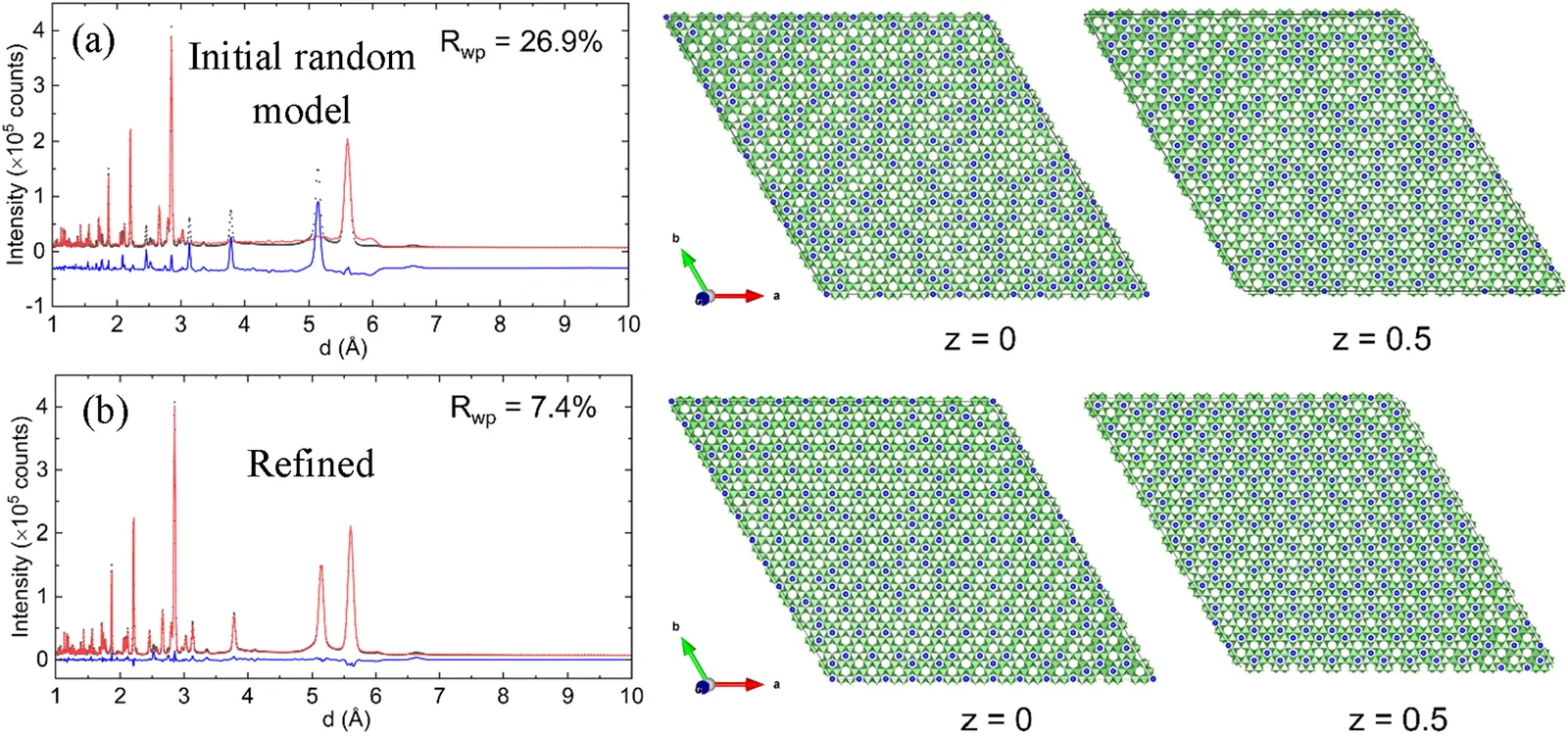
(a) Left: fit of the XRD data of Li_3_YCl_6_ cooled down to 40 °C (from 400 °C) with a randomly distributed Y-vacancy model. Right: (ab-plane) projection of the *z* = 0 and *z* = 0.5 planes of the model. The LiCl_6_ octahedra are shown in olive, Y is shown as blue spheres, and vacant sites are left empty. (b) Left: fit result of the XRD data after SA and convergence of the nonlinear least-squares refinements. Right: structure refined from the combined SA and nonlinear least-squares refinement.

Now, we shall have a comprehensive picture of the structure and phase transition mechanism of mechanically milled Li_3_YCl_6_ after different heat treatments. We also synthesized single-crystal Li_3_YCl_6_ by slowly cooling a melt from 800 to 200 °C at 3 °C/h, followed by annealing at 200 °C for 2 days. The refined crystal structure based on single-crystal X-ray diffraction adopts a trigonal symmetry with a space group of *P*3̅*m*1, as shown in Figure S22. Y fully occupies the 1a Wyckoff position and partially occupies two 2d sites, with occupancies of 12.0(3)% for the site in the same layer as 1a and 82.3(3)% for the site in the alternating layer (Table S13), which is similar to that in the highly crystalline powder sample and other previously reported results. The degree of Y disorder, defined by the occupancy of the 2d site in the layer alternating with the 1a site, is ∼12%. This is substantially lower than the 43% observed in mechanochemically synthesized samples (without annealing, Tables S4–S5), suggesting a strong dependence on the synthesis conditions. Li is found to occupy both the 6g and 6h sites within the Y layers, with refined occupancies of 83(2)% and 66(2)%, respectively, when the total Li content is constrained to 3 per formula unit. Notably, all single-crystal pieces examined by SXRD exhibit merohedral twinning related by a 60° rotation about [001], corresponding to the twin matrix $\left(\begin{array}{lll} 0 & -1 & 0\\ -1 & 0 & 0\\ 0 & 0 & -1 \end{array}\right)$ . The refined twin fraction of 0.508(3) indicates a nearly equal population of the two twinning domains. Representative diffraction zone images are provided in Figures S22a and S22b, showing well-indexed Bragg reflections. In the H0L zone, no 1D streaks due to stacking faults between layers are observed.

### Effect of Y-Vacancy Arrangements on the Li^+^ Conductivity

It has been reported that the Li^+^ conductivity of Li_3_YCl_6_ varies drastically among samples synthesized at different annealing temperatures. Many studies have reported higher RT Li^+^ ionic conductivity (0.2–1.0 mS/cm) in the ball-milled and 200 °C hot-pressed samples, while much lower Li^+^ conductivity of <0.05 mS/cm has been reported for the high-temperature-treated highly crystalline samples.^15,21,25^ To better understand how Y-vacancy ordering could affect the Li^+^ diffusion in the structure, we carried out kinetic Monte Carlo (KMC) analysis on several different ideal Y-vacancy ordering patterns and experimentally obtained structures. This approach enables the evaluation of Li^+^ diffusivity using realistic structural models, rather than the commonly employed idealized structural models (e.g., *P*3̅*m*1 or *Pnma*), which may not accurately capture the true structural characteristics. Furthermore, it allows the influence of long-range cation-vacancy ordering on Li^+^ diffusivity to be quantified, extending beyond the conventional assessment based solely on migration energy barriers calculated for idealized crystal structures.

The commonly used KMC approach for diffusion problem studies is used here, going beyond the time scale limitation of the MD simulation. A rejection-free approach is adopted, where at each step of the random walk of a single Li^+^ ion, an escape pathway is randomly selected according to its rate constant, and the residence time is drawn randomly from the exponential distribution associated with the rate constant. Specifically, we investigated the Li^+^ escape pathways within the layered LYC configuration, distinguishing between the in-plane (O–T–O) (Figure 13) and out-of-plane (O–O) pathways (Figure S23). It is well documented that the out-of-plane Li^+^ hopping (O–O) has much lower energy barriers relative to that of the in-plane pathways. Therefore, in-plane (O–T–O) diffusion should be the bottleneck for long-range Li^+^ diffusion within the LYC structure, and our analysis will focus on how in-plane Y-vacancy distributions affect Li^+^ hopping.^25,37^ The in-plane Li^+^ hopping pathways are further differentiated based on whether the neighboring LiCl_6_ octahedra are bridged by zero, one, or two YCl_6_ octahedra (Figure 13). In the Li^+^ hopping path (O–T–O) crossing the two YCl_6_ octahedra (noted as scenario 2 in Figure 13), the two intermediate tetrahedral sites above and below the Li layer (Figure 13) are both face-shared with the YCl_6_ octahedron and have a relatively high energy barrier (∼0.5 eV). For neighboring Li sites those are edge-shared with one Y (scenario 1), one of the O–T–O path (in this case the one above the Li layer, left side in Figure 13) has a high energy barrier (∼0.5 eV), while the other O–T–O path (the one below the Li layer, shown on the right side of Figure 13) has a much lower energy barrier (∼0.3 eV). For the case where the neighboring Li^+^ sites bridge the two empty octahedral sites (noted as scenario 0), both O–T–O hopping paths have a low energy barrier (∼0.3 eV).

**Figure 13:**
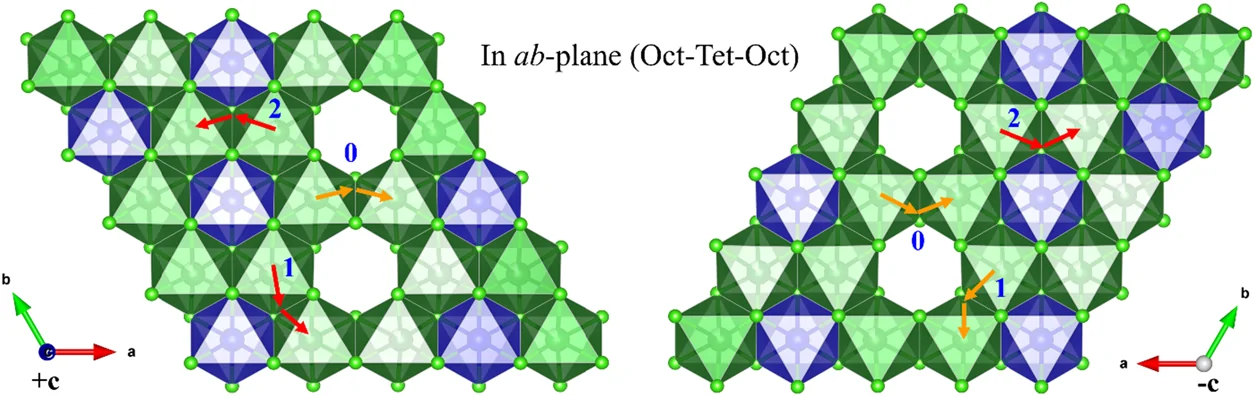
Illustration of in-plane (O–T–O) Li^+^ hopping pathways in the anion *hcp* close-packed Li_3_YCl_6_ (note: the Y-vacancy distribution in the *ab*-plane is intentionally chosen to show the three plausible different in-plane Li^+^ hopping scenarios). The left side shows the O–T–O pathways above the Li layer (positive *c*-axis direction), while the right side figure shows the O–T–O pathways below the Li layer (by flipping the left side figure by 180^◦^ around the *z*-axis).

Two different Y layers (*z* = 0 and *z* = 0.5) were analyzed separately, with a uniform set of energy barriers for Li^+^ hopping, i.e., 0.52 eV (the value reported by Qiu et al. is used for all KMC simulations presented here)^37^ for the O–T–O path that involves an intermediate tetrahedral site that is face-shared with the YCl_6_ octahedron (red arrows in Figure 13) and 0.27 eV for the O–T–O path that is faced-shared with the empty octahedral sites (orange arrows in Figure 13). As presented in Figures 14 and S24, the layer-1 (*z* = 0) and layer-2 (*z* = 0.5) in the ideal Y-vacancy honeycomb-ordered model show a “polarized” behavior—the diffusivity of layer-1 is the largest among all ideal Y-vacancy ordered models, whereas layer-2 shows the lowest diffusivity. This behavior arises from the uneven distribution of Y atoms between the two layers—layer-1 exhibits the minimum number of Y atoms sharing faces with the interstitial tetrahedral sites (Figure 3a, *z* = 0), whereas layer-2 (Figure 3a, *z* = 0.5) shows the opposite arrangement. The cross-square Y-vacancy-ordered model (Figure S7c) shows a similar “polarized” behavior, but the difference in the diffusivity between the two types of layers is much smaller compared to the honeycomb model (Figures 14 and S24). This is due to the more even distribution of Y cations between the *z* = 0 and *z* = 0.5 layers. By contrast, the herringbone, linear, three-line, and zigzag models all show a more balanced diffusivity between layer-1 (*z* = 0) and layer-2 (*z* = 0.5).

**Figure 14:**
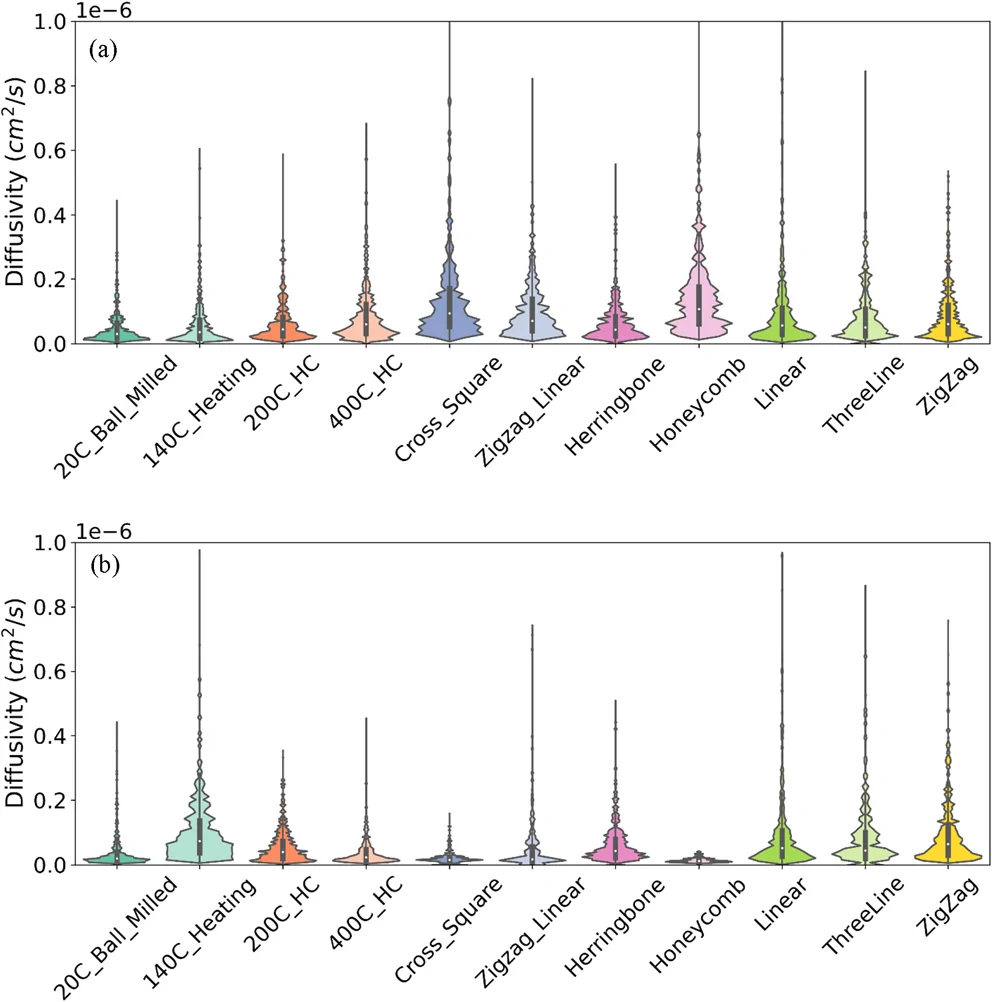
Violin plot shows the distribution of the in-plane diffusivity calculated from the KMC simulation for the theoretical configurations and those obtained from fitting against the experimental diffraction data. The results presented here are for layer-1 (*z* = 0) (a) and layer-2 (*z* = 0.5) (b). The horizontal width represents the probability density corresponding to a certain diffusivity value (the *y*-axis).

We then calculated Li^+^ diffusivity using structures obtained from the large-box structural refinements against the experimental diffraction data: the sample heated to 140 °C and the highly crystalline sample (heated to 400 °C and then cooled down to 40 °C) both show similar “polarized” Li^+^ diffuse behavior as that observed in the ideal Y-vacancy honeycomb-ordered model (Figure 4a), which is consistent with the fact that large regions of Y-vacancy honeycomb ordering are observed in the refined structures for both compounds (Figures 11 and 14). In contrast, the sample just after ball milling (Figure 9) shows similar Li^+^ diffusivity in both planes, which is likely associated with the presence of Y-rich and Y-poor regions in both planes, and the scattered distribution of these Y-rich regions may limit the long-range diffusion of Li^+^ in both planes. However, it is worth noting that the attempt frequencies are likely higher for Li^+^ ions residing in the Y-poor domains than those in the Y-rich domains. Therefore, the current KMC calculation of using a single prefactor (*p*_2_) represents a conservative estimate for scenario 0 in Figure 13. Specifically, the transition rates within the Y-poor domains are expected to be higher, which would lead to higher Li^+^ diffusivity in structures with higher concentrations of Y-poor domains across both planes, such as that in the ball-milled sample. The sample that shows the most balanced diffusion on both *z* = 0 and *z* = 0.5 planes is the sample heated to 200 °C, which has a significant portion of Y-vacancy zigzag-ordered regions with a balanced Y^3+^ distribution on the two different planes (ratio of 1.2). This Y-vacancy zigzag ordering-dominated phase is found to be stable between 160 and 240 °C (Figure 5), suggesting that a more balanced in-plane Li^+^ diffusivity can be achieved for samples synthesized in this temperature range (using the mechanically milled starting materials).

This observation is consistent with the measured RT Li^+^ conductivities (Table S14) and reports from other groups.^12,15,21^ It is worth noting that the Li^+^ ionic conductivities were all measured using relatively low-density pellets; therefore, the measured Li^+^ conductivities may not represent the real bulk Li-ionic conductivity because of the significant grain boundary contributions. Nevertheless, the much higher conductivities observed in the ball-milled and the 200 °C-treated samples (∼10^–4^ mS/cm), relative to those in the 140 °C-treated sample and the highly crystalline sample (400 °C-annealed) (∼10^–5^ mS/cm), suggest that a more homogeneous distribution of Y-vacancy across both planes is critical in achieving the optimized Li^+^ ionic conductivity in Li_3_YCl_6_. Similar behavior has also been observed in other anion hexagonal close-packed layered halide electrolytes, such as Li_3_ErCl_6_ and Li_3_YbCl_6_.^21,38,39^

This result indicates that variations in macroscopic ionic conductivity originate predominantly from differences in the connectivity of the Li^+^ transport network induced by the Y/vacancy distribution rather than from changes in the local hopping barriers themselves. This also suggests that designing more uniformly distributed RE-vacancy ordering patterns (both in- and out-of-plane), while deliberately increasing the number of vacancies (i.e., by aliovalent substitution of RE^3+^ with 4+ or 5+ cations), is critical for optimizing Li^+^ conductivity in *hcp* close-packed layered halide-based fast ionic conductors.

## Conclusions

Y^3+^ and vacancy distribution and ordering schemes can strongly affect the in-plane Li^+^ transport in Li_3_YCl_6_. We found that concentrating Y^3+^ on one layer, such as in a highly crystalline powder or single-crystal sample with ideal honeycomb ordering, leads to fast Li^+^ diffusivity in the Y^3+^-poor layer but very slow Li^+^ transport within the Y^3+^-rich layer. In contrast, having evenly distributed Y^3+^-vacancies on both planes, such as forming a Y-vacancy zigzag or linear-ordered structures, can facilitate fast Li^+^ transport in both planes. Moreover, it is found that the synthetic conditions can drastically affect the Y-vacancy distribution in the structure. The samples treated between 100 and 160 °C and those heated above 240 °C show large honeycomb-ordered Y-vacancy domains, with Y^3+^ unevenly distributed on the two layers, leading to very poor Li^+^ diffusivity within the Y-rich layer despite the facile Li^+^ transport in the Y-poor layer. A more balanced in-plane Li^+^ distribution has been identified for the ball-milled sample and samples heated between 160 and 240 °C. This more evenly distributed Y-vacancy leads to a more balanced Li^+^ diffusivity on both layers, which is likely the underlying reason for the observed higher Li^+^ conductivities. This study highlights the importance of manipulating Y-vacancy arrangements in optimizing Li^+^ conductivity in *hcp* close-packed halide-based fast Li^+^ conductors.

## Supplementary Material



## Notes

Experiment section: detailed data collection and structural analysis methods; additional figures on the structures and structural refinement results; DFT-calculated free energies of the structures with different defect types; additional Y-vacancy ordering patterns and refined large-box, structural models (DOCX)
