# Supplementary material for: Structure of Li3YCl6 and Its Effect on Lithium Ionic Conductivity
Source: J Am Chem Soc. 2026 Sep 17;148(38):40743–57. doi: 10.1021/jacs.6c06971 (PMC13632590; doi:10.1021/jacs.6c06971)
Supplement: Supplementary file 1 [file ja6c06971_si_001.docx]

**Structure of Li_3_YCl_6_ and Its Effect on Lithium Ionic Conductivity**

Jue Liu^a,*^, Yuanpeng Zhang^a^, Zhantao Liu^b^, Paul Cuillier^c,d^, Paul Cardon^e^, Megan Murphy^a^, Huiwen Ji^e^, Hailong Chen^b^

^a^ Neutron Scattering Division, Oak Ridge National Laboratory, Oak Ridge, 37831, TN, USA

^b^ Department of Mechanical Engineering, Georgia Institute of Technology, Atlanta, 30332, GA, USA

^c^ Department of Materials Science and Engineering, The Ohio State University, Columbus, 43210, OH, USA

^d^ Pritzker School of Molecular Engineering, University of Chicago, Chicago, 60637, IL, USA
^e^ Department of Materials Science and Engineering, The University of Utah, Salt Lake City, 84112, UT, USA

*Corresponding author email: [liuj1@ornl.gov](mailto:liuj1@ornl.gov)

**Experimental section**

**Materials synthesis**

All compounds were synthesized under Ar atmosphere. In a typical synthesis, ~0.8 g batches of mechanically milled Li_3_YCl_6_ were prepared by weighing anhydrous LiCl (>99%, Sigma-Aldrich) and anhydrous YCl_3_ (99.99%, Sigma-Aldrich) in a molar ratio of 3: 1 and ball-milled in zirconia jars (50 ml) with 10 zirconia balls (10 mm) at 500 revolutions per minute for 5h. For highly crystalline Li_3_YCl_6_, the ball-milled mixture was pressed into pellets and flame-sealed in a quartz tube, followed by a heating treatment at 400 ^o^C for 10h and successive quenching in air.

Single-crystal Li_3_YCl_6_ was synthesized by mixing and pelletizing stoichiometric LiCl (> 99%, Sigma Aldrich) and YCl_3_ (99.99%, Thermo Scientific Chemicals) in a sealed quartz ampule, which was brought to 800 °C at 1°C/min to melt the precursors and slowly cooled down to 200 °C at 3°C/hour following by annealing at 200 °C for two days.

**X-ray and neutron diffraction data collection and reduction**

In situ synchrotron X-ray powder diffraction (XRD) and total scattering data were collected at beam line 17-BM-B of the Advanced Photon Source (APS) at Argonne National Laboratory. XRD data were collected with an X-ray wavelength of 0.24117 Å and a sample-to-detector distance of 550 mm. X-ray total scattering data were collected with an X-ray wavelength of 0.24100 Å, a sample-to-detector distance of 210 mm, and a maximum momentum transfer *Q_max_* of 18 Å^-1^. The PDFGetX3 code^1^ was used to subtract the scattering signal of the empty quartz capillary and normalize the data to the expected composition Li_3_YCl_6_. Inelastic scattering contributions were corrected by enforcing the correct asymptotic limits for the structure function $S(Q)$, using $r_{poly}$ values of 0.96 Å and 1.08 Å for the ball milled and highly crystalline samples, respectively. The corrected $S(Q)$ were transformed to reduced pair distribution functions $G(r)$ with a sine Fourier transform:

$$\begin{aligned} G\left( r \right)=\frac{2}{\pi}\int_{Q_{min}}^{Q_{max}} Q\left[ S\left( Q \right)-1 \right]\sin\left( Qr \right)dQ (S1) \end{aligned}$$

Neutron powder diffraction and total scattering data were collected at the NOMAD instrument.^2, 3^ About 150 mg powder sample was loaded into 3 mm diameter thin-walled quartz capillary, an Oxford argon cryostream was used to control the temperature. The powder sample was first cooled down to 100 K and then ramped up and equilibrated at specific temperature for 10 min. Four 24 min scans were collected at each temperature point and summed together to improve the statistics. Neutron diffraction data were collected at 100 K, 200 K, 300 K, 400 K and 500 K. The collected diffraction data were background subtracted from the scattering signal of empty quartz capillary (collected at corresponding temperatures). Absorption corrections were carried out due to the relatively strong neutron absorption of natural Li and Cl. The background subtracted and absorption corrected scattering signal were then normalized to the scattering signal from a 6 mm diameter vanadium rod, to correct for detector efficiency.

For neutron pair distribution function data reduction. The pixel level-based absorption corrected neutron scattering data were merged into a single spectrum and then normalized by scattering signal from a 6 mm diameter vanadium rod. A two orders polynomial function was used to empirically fit for Placzek correction to obtain the arbitrarily normalized total scattering function S(Q). Sine Fourier transform was carried out using equation S1 with a Q_max_ cut of 30 Å^-1^. Fourier filtering was used to remove the uncounted multiple and inelastic scattering signals, and a near absolute g(r) was obtained by fine tuning the atomic density so that the g(r) is zero at any atomic distances that is smaller than 2 Å.

Single-crystal X-ray diffraction was collected on a Rigaku XtaLAB Synergy-R high-flux rotating anode diffractometer using a Mo source at 300 K.

**Structure refinement and data analysis methods**

Rietveld refinements and small box structure refinements using PDF data were carried out using TOPAS version 6.^4^ For structure refinements using XRD data, instrument parameter profile was obtained from refinement against a NIST Si 640d standard. Pseudo Voigt functions were used to model the crystallite size and microstrain broadening from the samples.

For structure refinements using neutron Bragg diffraction data. Time-of-flight (TOF) neutron diffraction data were converted to d-spacing data using a second order polynomial TOF = Zero + Difc*d + Difa*d^2^, where Zero is a constant, Difc is the diffraction constant. During the structure refinements, Zero and Difc were determined from refinement of standard NIST Si-640e data and held fixed, while Difa was allowed to vary to account for sample displacements. The instrument induced peak profile was modeled by convoluting a symmetrical Pseudo-Voigt function with a modified asymmetrical Ikeda-Carpenter-David function.^5-7^ Lorenz factor is corrected by multiplying (d_spacing)^4^.^8^

For small box structure refinement using neutron PDF data. A Lorentzian dampening function (Exp(-r*Q_damp_/2)) and Pseudo-Voigt-like peak profile were used to model the reduced G(r) data in TOPAS v6. An empirical (-δ/r^2^) term was used to describe the sharpening of low-r peaks due to the correlated atomic motion.^8^

Structure refinement using single crystal diffraction data was carried out using SHELXLE.^9^

**DFT calculation**

Density functional theory calculations were performed with Quantum ESPRESSO v7.3.1^10, 11^, using pslibrary v1.0.0^12^ projector-augmentor wave pseudopotentials and a PBE exchange correlation functional.^13^ Kinetic energy cutoffs were set to 70 Ry and 420 Ry for the wavefunction and charge density basis sets, respectively, to converge the accuracy of calculated total energies to within 1 meV/atom. Anti-site defect energies were calculated from variable-cell relaxations of $1\times1\times2$ supercells of the honeycomb $P\bar{3}m1$ structure, with a $2\times2\times2$ $\Gamma$-centered k-point mesh. Energies of the honeycomb, linear, trilinear, and zigzag Y vacancy ordering patterns were calculated with $\Gamma$ k-point sampling. To find the ground state Li distribution for each Y vacancy ordering, the occupancies of the possible Li sites (excluding ones that share a face with an occupied Y octahedra) were enumerated with Pymatgen.^14^ Then, for the 10 arrangements with the lowest electrostatic potential, energies were calculated with atoms at the average site positions, and the lowest energy configuration was selected for a variable-cell relaxation, with 50 meV/Å and 0.5 kbar convergence criteria for forces and stress, respectively.

**Supporting information S1**

Combined random walk Monte-Carlo and non-linear least square refinements of the large box model. As mentioned in the main text, non-linear least square refinements can be easily stuck in a local minimum during the reconstruction of complex Y-vacancy ordering patterns, such as that for the cross-square and mixed zigzag and linear ordering. Random walk Monte-Carlo allows the restart of the refinement using a newly generated randomly distributed Y-vacancy initial model. This is achieved by invoking the “continue_after_convergence” process in TOPAS.^4^ After each converged non-linear least square refinement (where the chi^2^_convergence_criteria is set to be 0.001), the occupancies of available Y sites are reset to either zero or one by a random generator (by using “val_on_continue = Round(Rand(0,1))” in TOPAS script). Typically, tens to hundreds of different randomly generated configurations are tested so that a global minimum can be encountered. A distance penalty function (Anti-bump) was used for bond length constraint of the nearest Y-Y pairs. Once a plausible global minimum is identified, a final cycle of non-linear least square refinement was carried out so that any slight deviations of Y site occupancies from 0 or 1 are round up, so that no partial occupancies are left on the available Y site. An example TOPAS input file of the combined random walk Monte-Carlo and non-linear leas square refinement is shown in the supporting information.

**Supporting information S2**

Combined simulated annealing and non-linear least square refinements of the large box model. Combined random walk MC and non-linear least square refinements were found to be not effective in reconstructing mixed zigzag and linear ordered and a few other complex ordered patterns. We developed a combined simulated annealing (SA) and non-linear least square refinement method to more effectively reconstruct the Y-vacancy arrangements. Similar to the structure solution using simulated annealing method.^15^ There are three steps for reconstructing the Y-vacancy arrangements from a Y-vacancy randomly distributed model. (1) Using non-linear least squares refinement to obtain the local minimum configuration by refining the occupancies of the available Y sites from the randomly distributed model. (2) Defining a temperature regime to simulate the annealing process. (3) Randomize the Y site occupancies according to the defined temperature regime. In the conventional simulated annealing process,^16^ random changes analogous to temperature are introduced into the cost function, and the acceptance of the new configuration is determined using Metropolis algorithm,^17^

Random (0,1) < P(ΔE), or Δ E < 0

Where ΔE is the change of energy from the previous states. Difficulties arise from determining an appropriate ΔE for this large system is obvious. Previous methods such as Reverse Monte Carlo use the changes of the refinement residual values between the current refinement and previous refinement to determine the probability, i.e., if the fit improves, the new configuration is accepted. If the fit becomes worse, there is a small probability that the new configuration will still be accepted.^18^ In our method, the nonlinear least squares refinement has been proven to be effective in reconstructing the Y-vacancy distribution from a randomly distribution model, which means that randomization process can accept any changes ranging from 0 to 1 for the Y-site occupancies. Thus, we can design a temperature scheme that, in theory, all scenarios in the acceptance test can be adopted, effectively remove the acceptance test. This has been previously used for the ab initio structure solution using simulated annealing method.^15^ In practice, we set there is 95% chance that the new configuration will be accepted, and the randomization of the Y-site occupancy is set to the previous refined value +/- Sqrt (0.2*T), and T ranges from 0.1 to 5. Any randomized value that is smaller than 0 or larger than 1 is reset to 0 and 1 respectively. There is 5% chance that the refined Y occupancies are accepted as starting values for the next refinement cycle. Once a likely global minimum is reached, the refined Y occupancies were adopted for the final cycle of nonlinear least squares refinement, where any partial occupancies on the Y site that are smaller than 0.1 is round up to 0 and any site occupancies that are larger than 0.9 is round up to 1.

**Supporting information S3**

The rejection-free Kinetic Monte Carlo (KMC) algorithm as detailed by A. F. Voter^19^ is taken here to perform the KMC simulation for the Li^+^ transportation. As detailed in the main text, there are three types of O-T-O pathways. For case-1, the tetrahedral sites (the ‘T’ in the pathway) above and below the Li-layer are both face-shared with a YCl_6_ octahedron. The energy barrier is taken as 0.52 eV (see the main text for the reference from which the energy barrier values were taken), with a pre-factor of 2 corresponding to the two tetrahedral sites above and below the Li-layer. For case-2, only one of the tetrahedral sites is face-shared with a YCl_6_ octahedron. In this case, the low-barrier pathway (i.e., the one without face-sharing with a YCl_6_ octahedron) is always taken – the energy barrier is taken as 0.27 eV and the pre-factor is 1. For case-3, none of the tetrahedral sites is face-shared with a YCl_6_ octahedron, in which case the energy barrier is still 0.27 eV but with a pre-factor of 2, since again, there are two alternative pathways through the tetrahedral site above or below the Li-layer.

For each transition event, the transition rate is calculated as,

$$k_{i}=p_{1}p_{2}\exp\left[ -\frac{E_{i}}{k_{B}T} \right] (1)$$

where $i$ is the event index, indicating one of the cases as detailed above. $p_{1}$ refers to the pre-factor described above, and $p_{2}$ is another pre-factor which is shared among all the different types of events. The $p_{2}$ factor is determined by the vibration frequencies of normal modes corresponding to the saddle points in the energy landscape and in principle would be different for different types of events. However, in practice, as followed in the current report, a common $p_{2}$ pre-factor can be assumed to be shared across different types of events, and here the value $p_{2}={10}^{12} s^{-1}$ is taken. The attempt frequencies are likely higher for Li^+^ ions residing in the Y-poor domains than in the Y-rich domains. Therefore, using a single pre-factor (*p_2_*) represents a conservative estimate for scenario 0 in Figure 13. Specifically, the transition rates within the Y-poor domains are expected to be higher, which would further increase the calculated Li^+^ diffusivity for structures with higher concentrations of Y-poor domains in both planes, such as that in the ball milled sample. $k_{B}$ refers to the Boltzmann constant and $T$ refers to temperature. The total transition rate is given as,

$$k_{tot}=\sum_{i} k_{i} (2)$$

from which the residence time at each KMC step is calculated as,

$$t=-\frac{1}{k_{tot}}\log\left( r \right) (3)$$

where $r$ refers to a random number on the interval $(0,1)$ generated at each KMC step. During the KMC simulation, the algorithm stochastically walks along the Li sites, and at each step of walking, an event (i.e., a specific O-T-O pathway) is picked according to the comparison of $r\cdot k_{tot}$ [again, $r$ refers to the random number generated at the current step, on the interval of $(0,1)$] against the cumulative transition rate stack (see Ref. ^19^ for a detailed explanation). Accordingly, the displacement (i.e., the distance between the two Li sites involved in the picked transition event) and residence time (Eqn. 3) could be obtained and accumulated. Such a KMC simulation could be repeated $N_{sample}$ times and for each time of the simulation, $N_{steps}$ steps could be performed. In the current report, $N_{sample}=1000$ and $N_{steps}=10000$ were taken for the simulation. Further, in the KMC simulation implemented in the current report, transitions are restricted to previously unvisited states to account only for the effective forward transport of Li atoms. The simulation terminates when any of the following conditions are met: (a) a preset limit of $N_{steps}$ steps is reached, (b) no unvisited states remain accessible, or (c) transitioning to an unvisited state becomes a rare event, defined here as an overall transition probability of less than 0.01 at a given Li site. For each of the $N_{sample}$ simulations, the diffusivity could be calculated from the slope of the squared displacement vs. the residence time, from which a probability distribution of the diffusivity could be obtained, as presented in Fig. 14 in the main text and Fig. S24 in the current SI.

(c)

(b)

(a)

(f)

(e)

(d)

**Figure S1.** Reported average structure of the highly crystalline Li_3_YBr_6_ (LYB) (top) and Li_3_YCl_6_ (LYC) (bottom). The former has the hexagonal close packed Br^-^ framework (ABCABC…) (**a**), the latter adopts the cubic close packed Cl^-^ framework (ABAB…) (**d**). In the well-ordered LYB structure (in S.G. *C*2/m), neighboring Y^3+^ layers are separated by a Li^+^ layer, and the minimum repeat sequence is three layers (A’B’C’…). On the Y layer, fully occupied Li and Y sites (1:2 ratio) form a honeycomb ordered pattern (**b-c**). In the well-ordered LYC structure (*S.G*. *P*$\bar{3}$m1), Y^3+^ occupies neighboring layers in a 1:2 ratio and the minimum repeat sequence is two layers (A’B’…). Y and Li sites form honeycomb ordering on each cation layer, but with different ratios between the two sites: for the Y-poor layer (**e**), the ratio between Y and Li sites is 1:2, while the ratio is opposite (2:1) for the Y-rich layer (**f**).

Neutron

Neutron

X-ray

X-ray

Neutron

Li-Cl

Y-Cl

**Figure S2.** Reduced X-ray PDF (Q_max_ = 18 Å^-1^) and neutron (Q_max_ = 30 Å^-1^) data of the highly crystalline Li_3_YCl_6_ and the phase just after ball milling. The wiggle around 3 Å in the X-ray PDF data is caused by the Fourier transform of S(Q) with limited Q_max_ (Q_max_ = 18 Å^-1^), as evidenced by the symmetrical wiggle on each side of the first G(r) peak. Both X-ray and neutron PDF data show that there are no noticeable differences in the G(r) data around 3 Å between the ball milled and highly crystalline samples (highlighted in green).

**Figure S3.** Energy calculated with density functional theory for several different Y-vacancy defects in the Li_3_YCl_6_. All energies are relative to the ground state honeycomb ordering shown in (a). (b) Model with all Y occupying a single cation layer (either z = 0 or z = 0.5). (c) Anti-site defect with a single pair of edge sharing YCl_6_ octahedra. (d) Anti-site defect with a triplet of corner-sharing YCl_6_ octahedra. (e) Defect with a pair of face-sharing YCl_6_ octahedra. (f) Stacking fault model that creates a triplet of face-sharing YCl_6_ octahedra.

**Figure S4.** Structure refinements of ball milled Li_3_YCl_6_ (before heating) against neutron diffraction data, with anti-site defect model enabled.

**Figure S5.** Structure refinements of Li_3_YCl_6_ (highly crystalline) against neutron diffraction data, with Li-Y anti-site defects enabled.

**Figure S6.** Rietveld refinements of ball milled Li_3_YCl_6_ (left, before heating), and highly crystalline Li_3_YCl_6_ against XRD data (right), with anti-site defect model enabled.

(a)

(b)

(c)

(d)

(e)

**Figure S7.** Different Y-vacancy ordering patterns: (**a**) three-line ordering; (**b**) mixing zig-zag and linear ordering; (**c**) cross square ordering; (**d**) and **(e)** random distributed Y/vacancy models. The left side shows the cation and vacancy distribution on the C’ layer (*z* = 0), the right side shows the C’’ layer (*z* = 0.5).

**Figure S8.** Comparison between the simulated XRD (S.G. *P*$\bar{3}$m1) and the measured XRD data of the ball milled Li_3_YCl_6_ at 20 $^{\circ}$C (before heating).

**Figure S9.** Comparison between the simulated XRD (S.G. *P*nma) and the measured XRD data of Li_3_YCl_6_ heated to 220 $^{\circ}$C. Note: 20 $^{\circ}$C lattice parameters were used for the simulation; therefore, the simulated Bragg reflections appear at smaller d-spacings.

**Figure S10.** Compare the in situ XRD data collected at 400 $^{\circ}$C, 320 $^{\circ}$C (during cooling), 200 $^{\circ}$C (during cooling) and 40 $^{\circ}$C(cooling). The broadening of Y-vacancy ordered superlattice peaks are obvious when samples are cooled down below 320 $^{\circ}$C.

**Figure S11.** The small amounts of impurities (highlighted) formed in Li_3_YCl_6_ when the sample is heated to above 320 °C. Intensities of Bragg reflections associated with the impurity increase as the temperature increases above 320 °C, while peak positions of the associated reflections are much unchanged. This is in clear contrast to the main phase (Li_3_YCl_6_), which shows relatively large thermal expansions as the Bragg peaks continuously shift toward larger d-spacing as temperature increases.

(b)

(a)

(d)

(c)

**Figure 12.** Rietveld refinements of structures of Li_3_YCl_6_ during in situ heating experiments (20 °C, 140 °C, 220 °C and 320 °C). The starting materials are the ball milled LiCl and YCl_3_.

**Figure S13.** Rietveld refinement of the structure of sample heated to 220 C against XRD data. The Li-Y zig-zag ordered model (S.G. *P*nma) is used for the structure refinement.

**Figure S14.** Rietveld refinement of the structure (S.G. *P*$\bar{3}$m1) of the ball milled sample against neutron diffraction data. Data were collected from NOMAD at 300 K.

**Figure S15.** Rietveld refinement of the structure (S.G. *P*nma) of ball milled sample after heating at 500 K. Data was collected at NOMAD instrument.

(a)

(b)

(c)

**Figure S16.** Reconstruction of linear (a), threeline (b), and herringbone (c) type Y-vacancy ordering from randomly distributed Y-vacancy distribution models.

(a)

(b)

**Figure S17.** Failed reconstructions of the Y-vacancy cross-square (R_wp_ =4.3%) (a) and the mixed zig-zag and linear (R_wp_ = 1.8%) (b) ordering configuration using Rietveld refinements from one randomly distributed Y-vacancy starting models.

(a)

(b)

**Figure S18.** (a) Reconstruction of the cross-square Y-vacancy ordering model from Y-vacancy randomly distributed models using combined random-walk Monte Carlo and non-linear least-squares refinements. Top figure shows the R_wp_ change as a fucntion of refinement iterations. Bottom figure shows the final converged result with cross-square Y-vacancy ordering successfully reconstructed. (b) Reconstruction of the mixed zigzag and linear Y-vacancy ordering after tens of refinement cycles using random-walk Monte Carlo and nonlinear least-squares method.

(a)

(b)

**Figure S19.** Success reconstruction of the mixed zigzag and linear Y-vacancy ordering model (a), cross-square ordering model (b), Herringbone ordering (c), and honeycomb ordering (d) from initial Y-vacancy randomly distributed models using combined simulated annealing (SA) and non linear least-squares refinement method.

(b)

(a)

(d)

(c)

**Figure 20.** Co-refinement results using XRD (a), neutron diffraction (b) and neutron pair distribution function data (c) without refining the atomic coordinates of Cl. (d) Zoom in of the misfit of the short-range neutron PDF (1.5 – 10 Å). The most noticeable discrepancies arise from peaks around 2.6 Å and 3.8 Å, corresponding to Li-Cl/Y-Cl bonds and nearest Cl-Cl pairs in the structure.

(b)

(a)

**Figure S21.** (a) Left, fit of the XRD data of Li_3_YCl_6_ heated to 320 °C with the randomly distributed Y-vacancy model. Right, (ab-plane) projection of the z= 0 and z =0.5 planes of the model. LiCl_6_ octahedra are shown in olive, Y are shown as blue spheres, and vacant sites are left empty. (b) Left, fit result of the XRD data after simulated annealing and convergence of the least square refinements. Right, structure refined from the combined SA and least square refinement.

(b)

(a)

(c)

**Figure S22.** Non-symmetrized single-crystal X-ray diffraction zone images of (a) *HK*0 and (b) *H*0*L*. (c) The refined structure from single crystal Li_3_YCl_6_.

**Figure S23.** Illustration of out-of-plane (O-O) Li^+^ hopping pathways in the anion *hcp* close packed Li_3_YCl_6_.

(b)

(a)

**Figure S24.** The in-plane diffusivity calculated from the KMC simulation for theoretical configurations and those obtained from fitting against the experimental neutron diffraction data. (a) layer-1 with z = 0 (b) layer-2 with z = 0.5.

we carried out quantitative stacking-fault modeling using the ideal honeycomb-ordered Y/vacancy structure (S.G. *P*$\bar{3}$m1) as the starting model. The simulation results are shown in **Figures S25** and **S26**. As can be seen, the stacking-fault model is only able to partially reproduce the broad diffuse scattering observed for samples heat-treated below 160 °C. The fit quality deteriorates significantly for samples treated between 160 and 240 °C, where it fails to reproduce the diffuse superlattice reflections associated with the change of Y-vacancy ordering patterns (**Figure S26**).

More importantly, refinements based solely on the ideal honeycomb-ordered Y/vacancy model results in sharp Bragg reflections corresponding to the in-plane Y-vacancy ordering, such as the (2-11) and (3-1-1) reflections (**Figure S25**), in clear disagreement with the experimental diffraction patterns. To reproduce the observed diffuse scattering, it is necessary to introduce site mixing between the Y2 and Y3 sites, with a refined site-mixing fraction of approximately 33%. This result demonstrates that disorder in the in-plane Y-vacancy distribution is required to account for the experimental diffraction features. Together, these findings provide strong evidence that the Y-vacancy distribution is the primary origin of the diffuse scattering in the ball-milled Li_3_YCl_6_ samples.

(a)

(b)

**Figure S25.** Stacking-fault modeling of the diffuse scattering observed in the X-ray diffraction pattern of the ball-milled Li3YCl6 sample. (a) Simulation using an ideal honeycomb-ordered Y/vacancy model, i.e., occ_Y1 and occY3 are set to be one while occ_Y2 is set to zero (see **Figure 1** in the main text). Although introducing cation stacking disorder partially reproduces the diffuse scattering, the model also generates sharp Bragg reflections associated with the in-plane Y-vacancy ordering (e.g., 2-11 and 3-11 reflections in S.G. *P*$\bar{3}$m1). (b) To broaden these sharp Bragg reflections into the experimentally observed diffuse scattering, site mixing between the Y2 and Y3 sites must be introduced. This site mixing is equivalent to the non-ideal Y-vacancy distribution incorporated in our large-box model (**Figure 8**), demonstrating that disorder in the in-plane Y-vacancy arrangement is required to explain the diffuse scattering feature the in the ball milled sample.

(a)

(b)

**Figure S26.** Stacking-fault modeling of the X-ray diffraction pattern of the sample heat-treated at 200 °C. (a) Simulation using an ideal honeycomb-ordered Y/vacancy model, in which occ_Y1 and occY3 are set to be one while occ_Y2 is set to zero (see Figure 1 in the main text). The stacking-disordered model based on the honeycomb-ordered Y/vacancy arrangement provides only a poor fit to the broad superlattice reflections (highlighted), indicating that stacking disorder is unlikely to be the dominant defect in the 200 °C heat-treated sample. As discussed in the main text, cation migration and the associated redistribution of Y vacancies are likely responsible for the observed phase transition near 160 °C (**Figure 5**).

**Table S1.** Rietveld refinements of ball milled Li_3_YCl_6_ (before heating) against XRD data, with anti-site defect model enabled (293.5 K, 20 °C).

| Ball milled Li_3_YCl_6_ (293.5 K), XRD data (λ = 0.24117 Å)  Space group *P*$\bar{3}$m1, a = b = 11.1955(12) Å, c = 6.0394(9) Å; R_wp_ = 7.48%, Gof = 9.92 | | | | | | |
| --- | --- | --- | --- | --- | --- | --- |
|  | Wyck. | x | y | z | occ | Beq (Å^2^) |
| Y1 | 1a | 0 | 0 | 0 | 0.81(2)** | 3.0(5) |
| Y2 | 2d | 1/3 | 2/3 | 0 | 0.41(1)** | 1.2(3) |
| Y3 | 2d | 1/3 | 2/3 | 0.5 | 0.58(1)** | 1.2(3) |
| Y4 | 1a | 0 | 0 | 0.5 | 0.09(2)** | 3.0(5) |
| Cl1 | 6i | 0.1074(9) | -0.1074(9) | 0.8004 (20) | 1 | 3.0(5) |
| Cl2 | 6i | 0.2205(11) | -0.2205(11) | 0.2610(22) | 1 | 1.2(5) |
| Cl3 | 6i | 0.4482(10) | -0.4482(10) | 0.7517(21) | 1 | 0.8(4) |
| Li1 | 6g | 0.345(14) | 0 | 0 | 0.68(14)** | 4.3* |
| Li2 | 6h | 0.345(11) | 0 | 0.5 | 0.85(12)** | 4.3* |

**constraint: the max of Y1 occ is set to be 1- occY4; the max of Y2 occ is set to be 1-occY3. **For Li sites, the occ constraint is set to be min = 0 and max =1.

*fixed

**Table S2.** Rietveld refinements of ball milled Li_3_YCl_6_ (before heating) against neutron diffraction data (300 K), with anti-site defect model enabled.

| Ball milled Li_3_YCl_6_ (300 K), TOF neutron data from NOMAD  Space group *P*$\bar{3}$m1, a = b = 11.2046(3) Å, c = 6.0398(2) Å; R_wp_ = 1.84%, Gof = 1.63 | | | | | | |
| --- | --- | --- | --- | --- | --- | --- |
|  | Wyck. | x | y | z | occ | Beq (Å^2^) |
| Y1 | 1a | 0 | 0 | 0 | 0.73(5)** | 2.06(26) |
| Y2 | 2d | 1/3 | 2/3 | 0 | 0.45(1)** | 0.09(7) |
| Y3 | 2d | 1/3 | 2/3 | 0.5 | 0.54(3)** | 0.09(7) |
| Y4 | 1a | 0 | 0 | 0.5 | 0.26(7)** | 2.06(26) |
| Cl1 | 6i | 0.1134(3) | -0.1134(3) | 0.7736(7) | 1 | 2.44(10) |
| Cl2 | 6i | 0.2220(3) | -0.2220(3) | 0.2640(7) | 1 | 1.44(11) |
| Cl3 | 6i | 0.4461(3) | -0.4461(3) | 0.7518(7) | 1 | 1.27(10) |
| Li1 | 6g | 0.3235(27) | 0 | 0 | 0.93(9)** | 4.3* |
| Li2 | 6h | 0.3237(54) | 0 | 0.5 | 0.62(9)** | 4.3* |

**constraint: the max of Y1 occ is set to be (1- occY4); the max of Y2 occ is set to be (1-occY3). **For Li sites, the occ constraint is set to be min = 0 and max =1.

*fixed

**Table S3.** Rietveld refinements of highly crystalline (quenched after annealing at 400°C) Li_3_YCl_6_ against neutron diffraction data, with anti-site defect model enabled.

| Ball milled Li_3_YCl_6_ (300 K), TOF neutron data from NOMAD  Space group P$\bar{3}$m1, a = b = 11.2087(3) Å, c = 6.0491(2) Å; R_wp_ = 2.10%, Gof = 2.31 | | | | | | |
| --- | --- | --- | --- | --- | --- | --- |
|  | Wyck. | x | y | z | occ | Beq (Å^2^) |
| Y1 | 1a | 0 | 0 | 0 | 0.95(1)** | 3.25(28) |
| Y2 | 2d | 1/3 | 2/3 | 0 | 0.13(3)** | 0.13(7) |
| Y3 | 2d | 1/3 | 2/3 | 0.5 | 0.86(2)** | 0.13(7) |
| Y4 | 1a | 0 | 0 | 0.5 | 0.05(2)** | 3.25(28) |
| Cl1 | 6i | 0.1134(4) | -0.1134(4) | 0.7661(15) | 1 | 2.06(14) |
| Cl2 | 6i | 0.2206(4) | -0.2206(4) | 0.2713(13) | 1 | 1.64(14) |
| Cl3 | 6i | 0.4463(4) | -0.4463(4) | 0.7568(11) | 1 | 1.23(12) |
| Li1 | 6g | 0.2937(31) | 0 | 0 | 0.56(4)** | 3.3* |
| Li2 | 6h | 0.3187(19) | 0 | 0.5 | 1.00(5)** | 3.3* |

**constraint: the max of Y1 occ is set to be (1- occY4); the max of Y2 occ is set to be (1-occY3). **For Li sites, the occ constraint is set to be min = 0 and max =1.

*fixed

**Table S4** Rietveld refined structure of ball milled Li_3_YCl_6_ (before heating) against neutron diffraction data (300 K).

| Ball milled Li_3_YCl_6_ (300 K), TOF neutron data from NOMAD  Space group *P*$\bar{3}$m1, a = b = 11.2046(3) Å, c = 6.0398(2) Å; R_wp_ = 1.85%, Gof = 1.64 | | | | | | |
| --- | --- | --- | --- | --- | --- | --- |
|  | Wyck. | x | y | z | occ | Beq (Å^2^) |
| Y1 | 1a | 0 | 0 | 0 | 0.85(3) | 3.16(26) |
| Y2 | 2d | 1/3 | 2/3 | 0 | 0.44(2) | 0.31(7) |
| Y3 | 2d | 1/3 | 2/3 | 0.5 | 0.56(2) | 0.31(7) |
| Y4 | 1a | 0 | 0 | 0.5 | 0.15(3) | 3.16(26) |
| Cl1 | 6i | 0.1133(3) | -0.1133(3) | 0.7741(7) | 1 | 2.52(10) |
| Cl2 | 6i | 0.2218(3) | -0.2218(3) | 0.2619(7) | 1 | 1.49(10) |
| Cl3 | 6i | 0.4460(3) | -0.4460(3) | 0.7495(7) | 1 | 1.33(10) |
| Li1 | 6g | 0.3246(35) | 0 | 0 | 0.83(9) | 4.33(16) |
| Li2 | 6h | 0.3327(51) | 0 | 0.5 | 0.67(9) | 4.32(15) |

**Table S5** Rietveld refined structure of ball milled Li_3_YCl_6_ (before heating) against XRD data (293.5 K, 20 °C).

| Ball milled Li_3_YCl_6_ (293.5 K), XRD data (λ = 0.24117 Å)  Space group *P*$\bar{3}$m1, a = b = 11.1955(12) Å, c = 6.0395(9) Å; R_wp_ = 7.49%, Gof = 9.93 | | | | | | |
| --- | --- | --- | --- | --- | --- | --- |
|  | Wyck. | x | y | z | occ | Beq (Å^2^) |
| Y1 | 1a | 0 | 0 | 0 | 0.83(1) | 3.0(5) |
| Y2 | 2d | 1/3 | 2/3 | 0 | 0.41(1) | 1.5(2) |
| Y3 | 2d | 1/3 | 2/3 | 0.5 | 0.59(1) | 1.5(2) |
| Y4 | 1a | 0 | 0 | 0.5 | 0.17(1) | 3.0(5) |
| Cl1 | 6i | 0.1070(8) | -0.1070(8) | 0.7998(20) | 1 | 2.0(4) |
| Cl2 | 6i | 0.2208(11) | -0.2208(11) | 0.2615(23) | 1 | 1.3(5) |
| Cl3 | 6i | 0.4481(10) | -0.4481(10) | 0.7517(22) | 1 | 0.8(3) |
| Li1 | 6g | 0.341(14) | 0 | 0 | 0.68(11) | 4.3* |
| Li2 | 6h | 0.345(11) | 0 | 0.5 | 0.82(11) | 4.3* |

***** fixed to the values refined from neutron diffraction data

**Table S6** Rietveld refined structure of highly crystalline Li_3_YCl_6_ against neutron diffraction data (300 K).

| Ball milled Li_3_YCl_6_ (300 K), TOF neutron data from NOMAD  Space group P$\bar{3}$m1, a = b = 11.2088(3) Å, c = 6.0492(2) Å; R_wp_ = 2.10%, Gof = 2.31 | | | | | | |
| --- | --- | --- | --- | --- | --- | --- |
|  | Wyck. | x | y | z | occ | Beq (Å^2^) |
| Y1 | 1a | 0 | 0 | 0 | 0.97(3) | 3.23(30) |
| Y2 | 2d | 1/3 | 2/3 | 0 | 0.14(2) | 0.14(8) |
| Y3 | 2d | 1/3 | 2/3 | 0.5 | 0.86(2) | 0.14(8) |
| Y4 | 1a | 0 | 0 | 0.5 | 0.03(3) | 3.23(30) |
| Cl1 | 6i | 0.1133(4) | -0.1133(4) | 0.7658(17) | 1 | 2.01(15) |
| Cl2 | 6i | 0.2205(4) | -0.2205(4) | 0.2713(15) | 1 | 1.55(14) |
| Cl3 | 6i | 0.4460(3) | -0.4460(3) | 0.7568(14) | 1 | 1.26(13) |
| Li1 | 6g | 0.2952(56) | 0 | 0 | 0.50(9) | 2.47(80) |
| Li2 | 6h | 0.3196(30) | 0 | 0.5 | 1.00(9) | 3.29(50) |

**Table S7** Rietveld refined structure of ball milled Li_3_YCl_6_ against neutron diffraction data (500 K).

| Ball milled Li_3_YCl_6_ heated to 500 K, TOF neutron data from NOMAD  Space group *P*nma, a = 13.0062(9) Å, b = 11.26326(8) Å, c = 6.1442(2) Å  R_wp_ = 1.63%, Gof = 4.11 | | | | | | |
| --- | --- | --- | --- | --- | --- | --- |
|  | Wyck. | x | y | z | Occ | Beq (Å^2^) |
| Y1 | 4 | 0.3819(6) | 0.25 | 0.0122(12) | 0.83(1) | 1.32(7) |
| Y2 | 4 | 0.3781(31) | 0.25 | 0.5450(36) | 0.17(1) | 1.32(7) |
| Cl1 | 8 | 0.7890(4) | 0.4165(5) | 0.7124(3) | 1 | 2.86(9) |
| Cl2 | 4 | 0.4545(5) | 0.75 | 0.2360(7) | 1 | 1.46(9) |
| Cl3 | 8 | 0.4658(4) | 0.4149(5) | 0.2558(5) | 1 | 2.26(8) |
| Cl4 | 4 | 0.2100(6) | 0.25 | 0.2441(6) | 1 | 2.69(12) |
| Li1 | 8 | 0.8813(40) | 0.5983(54) | 0.5* | 0.58(3) | 4.99(30) |
| Li2 | 8 | 0.6021(22) | 0.4397(30) | 0.5* | 0.92(3) | 4.99(30) |

***** fixed

**Table S8** Rietveld refined structure of ball milled Li_3_YCl_6_ against in situ XRD data collected at 140 °C (S.G. P-3m1)

| Ball milled Li_3_YCl_6_ (140 °C), XRD data (λ = 0.24117 Å)  Space group *P*$\bar{3}$m1, a = b = 11.1829(7) Å, c = 6.03078(5) Å; R_wp_ = 11.1%, Gof = 13.9 | | | | | | |
| --- | --- | --- | --- | --- | --- | --- |
|  | Wyck. | x | y | z | occ | Beq (Å^2^) |
| Y1 | 1a | 0 | 0 | 0 | 0.95(2) | 3.9(5) |
| Y2 | 2d | 1/3 | 2/3 | 0 | 0.35(1) | 1.2(2) |
| Y3 | 2d | 1/3 | 2/3 | 0.5 | 0.65(1) | 1.2(2) |
| Y4 | 1a | 0 | 0 | 0.5 | 0.05(2) | 3.9(5) |
| Cl1 | 6i | 0.1103(20) | -0.1103(20) | 0.7803(36) | 1 | 2.7(1) |
| Cl2 | 6i | 0.2208(21) | -0.2208(21) | 0.2708(39) | 1 | 2.7(1) |
| Cl3 | 6i | 0.4440(20) | -0.4440(20) | 0.7558(35) | 1 | 2.7(1) |
| Li1 | 6g | 0.3333* | 0 | 0 | 0.68(19) | 4* |
| Li2 | 6h | 0.3333* | 0 | 0.5 | 0.82(19) | 4* |

***** fixed

**Table S9** Rietveld refined structure of ball milled Li_3_YCl_6_ against in situ XRD data collected at 220 °C (S.G. Pnma)

| Ball milled Li_3_YCl_6_ (220 °C), XRD data (λ = 0.24117 Å)  Space group *P*nma, a = 13.0323(29) Å, b = 11.2866(26) Å, c = 6.1751(6) Å  R_wp_ = 11.87%, Gof = 14.96 | | | | | | |
| --- | --- | --- | --- | --- | --- | --- |
|  | Wyck. | x | y | z | Occ | Beq (Å^2^) |
| Y1 | 4 | 0.3796(9) | 0.25 | -0.0076(23) | 0.75(1) | 2.2(2) |
| Y2 | 4 | 0.3806(29) | 0.25 | 0.5469(43) | 0.25(1) | 2.2(2) |
| Cl1 | 8 | 0.7802(15) | 0.4005(15) | 0.7205(23) | 1 | 3.7(6) |
| Cl2 | 4 | 0.4572(19) | 0.75 | 0.2245(27) | 1 | 2.1(7) |
| Cl3 | 8 | 0.4652(16) | 0.4226(15) | 0.2468(24) | 1 | 3.0(5) |
| Cl4 | 4 | 0.2143(22) | 0.25 | 0.2344(30) | 1 | 3.5(9) |
| Li1 | 8 | 0.870(20) | 0.573(20) | 0.5* | 0.5(7) | 5* |
| Li2 | 8 | 0.645(8) | 0.457(8) | 0.5* | 1.0(7) | 5* |

***** fixed

**Table S10** Rietveld refined structure of ball milled Li_3_YCl_6_ against in situ XRD data collected at 320 °C (S.G. *P*$\bar{3}$m1)

| Ball milled Li_3_YCl_6_ (320 °C), XRD data (λ = 0.24117 Å)  Space group *P*$\bar{3}$m1, a = b = 11.3481(3) Å, c = 6.2515 (2) Å; R_wp_ = 5.05%, Gof = 6.57 | | | | | | |
| --- | --- | --- | --- | --- | --- | --- |
|  | Wyck. | x | y | z | occ | Beq (Å^2^) |
| Y1 | 1a | 0 | 0 | 0 | 1.00(1) | 2.4(2) |
| Y2 | 2d | 1/3 | 2/3 | 0 | 0.22(1) | 4.7(2) |
| Y3 | 2d | 1/3 | 2/3 | 0.5 | 0.88(1) | 4.7(2) |
| Y4 | 1a | 0 | 0 | 0.5 | 0.00(1) | 2.4(2) |
| Cl1 | 6i | 0.1131(5) | -0.1131(5) | 0.7861(8) | 1 | 5.0(1) |
| Cl2 | 6i | 0.2205(7) | -0.2205(7) | 0.2651(9) | 1 | 5.0(1) |
| Cl3 | 6i | 0.4390(5) | -0.4390(5) | 0.7722(9) | 1 | 5.0(1) |
| Li1 | 6g | 0.3333* | 0 | 0 | 0.50(4) | 5.0* |
| Li2 | 6h | 0.3333* | 0 | 0.5 | 1.00(4) | 5.0* |

***** fixed

**Table S11** Rietveld refined structure of ball milled Li_3_YCl_6_ against in situ XRD data collected at 400 °C (S.G. *P*$\bar{3}$m1)

| Ball milled Li_3_YCl_6_ (400 °C), XRD data (λ = 0.24117 Å)  Space group *P*$\bar{3}$m1, a = b = 11.3995(4) Å, c = 6.3145(3) Å; R_wp_ = 6.42%, Gof = 8.35 | | | | | | |
| --- | --- | --- | --- | --- | --- | --- |
|  | Wyck. | x | y | z | occ | Beq (Å^2^) |
| Y1 | 1a | 0 | 0 | 0 | 0.99(1) | 2.4(3) |
| Y2 | 2d | 1/3 | 2/3 | 0 | 0.08(1) | 4.8(2) |
| Y3 | 2d | 1/3 | 2/3 | 0.5 | 0.92(1) | 4.8(2) |
| Y4 | 1a | 0 | 0 | 0.5 | 0.01(1) | 2.4(3) |
| Cl1 | 6i | 0.1113(7) | -0.1113(7) | 0.7775(16) | 1 | 5.0(1) |
| Cl2 | 6i | 0.2190(7) | -0.2190(7) | 0.2778(18) | 1 | 5.0(1) |
| Cl3 | 6i | 0.4363(5) | -0.4363(5) | 0.7667(16) | 1 | 5.0(1) |
| Li1 | 6g | 0.3333* | 0 | 0 | 0.50(8) | 5.0* |
| Li2 | 6h | 0.3333* | 0 | 0.5 | 1.00(8) | 5.0* |

***** fixed

**Table S12** Rietveld refined structure of ball milled Li_3_YCl_6_ against in situ XRD data (first heated to 400 °C and then cooled down to 40 °C, S.G. *P*$\bar{3}$m1)

| Ball milled Li_3_YCl_6_ (40 °C), XRD data (λ = 0.24117 Å)  Space group *P*$\bar{3}$m1, a = b = 11.1872(5) Å, c = 6.0501(3) Å; R_wp_ = 8.58%, Gof = 10.96 | | | | | | |
| --- | --- | --- | --- | --- | --- | --- |
|  | Wyck. | x | y | z | occ | Beq (Å^2^) |
| Y1 | 1a | 0 | 0 | 0 | 0.99(1) | 1.4(2) |
| Y2 | 2d | 1/3 | 2/3 | 0 | 0.17(2) | 1.2(1) |
| Y3 | 2d | 1/3 | 2/3 | 0.5 | 0.83(2) | 1.2(1) |
| Y4 | 1a | 0 | 0 | 0.5 | 0.01(1) | 1.4(2) |
| Cl1 | 6i | 0.1130(10) | -0.1130(10) | 0.7717(20) | 1 | 1.96(6) |
| Cl2 | 6i | 0.2187(12) | -0.2187(12) | 0.2648(20) | 1 | 1.96(6) |
| Cl3 | 6i | 0.4440(10) | -0.4440(10) | 0.7586(20) | 1 | 1.96(6) |
| Li1 | 6g | 0.325(7) | 0 | 0 | 0.82(13) | 4.3* |
| Li2 | 6h | 0.312(11) | 0 | 0.5 | 0.68(13) | 4.3* |

***** fixed

**Table S13.** Crystallographic parameters for single crystal Li_3_YCl_6_.

| chemical formula | | | | | | | Li_2.98(8)_Y_0.962(4)_Cl_6_ | | | | | | | |
| --- | --- | --- | --- | --- | --- | --- | --- | --- | --- | --- | --- | --- | --- | --- |
| crystal system | | | | | | | trigonal | | | | | | | |
| space group | | | | | | | *P*$\bar{3}$*m1* (#164) | | | | | | | |
| *a* (Å) | | | | | | | 11.1866(4) | | | | | | | |
| *c* (Å) | | | | | | | 6.0358(2) | | | | | | | |
| *V* (Å^3^) | | | | | | | 654.13(6) | | | | | | | |
| *Z* | | | | | | | 3 | | | | | | | |
| radiation | | | | | | | Mo K𝛼, 𝜆 = 0.71073 Å | | | | | | | |
| temp. (K) | | | | | | | 300 | | | | | | | |
| abs. coeff. (mm^-1^) | | | | | | | 8.209 | | | | | | | |
| *F*(0 0 0) | | | | | | | 446 | | | | | | | |
| reflections collected: total/unique | | | | | | | 5067/709 | | | | | | | |
| unique/internal data agreement | | | | | | | R_int_ = 0.0231 | | | | | | | |
| data/param | | | | | | | 709/42 | | | | | | | |
| GOF | | | | | | | 1.210 | | | | | | | |
| *R* | | | | | | | 0.0236 | | | | | | | |
| *R_w_* (*F*^2^) | | | | | | | 0.0387 | | | | | | | |
| largest difference peak/hole (Å^-3^) | | | | | | | 0.464/-0.424 | | | | | | | |
|  | *x* | | *y* | | *z* | | | occ. | | *U*_iso_ (Å^2^) | | site | | |
| Y1 | –0.333333 | | 0.333333 | | –0.4869(4) | | | 0.823(3) | | 0.0119(2) | | 2d | | |
| Y2 | –0.333333 | | 0.333333 | | –0.005(4) | | | 0.120(3) | | 0.034(3) | | 2d | | |
| Y3 | 0.000000 | | 0.000000 | | 0.000000 | | | 1.000 | | 0.0190(3) | | 1a | | |
| Cl4 | -0.22111(9) | | 0.22111(9) | | -0.73456(19) | | | 1.000 | | 0.0306(5) | | 6i | | |
| Cl5 | 0.11475(8) | | 0.22949(16) | | 0.23325(18) | | | 1.000 | | 0.0278(4) | | 6i | | |
| Cl6 | -0.44612(9) | | 0.10776(17) | | -0.24462(15) | | | 1.000 | | 0.0218(4) | | 6i | | |
| Li7 | -0.3426(9) | | 0.000000 | | 0.000000 | | | 0.83(2) | | 0.082(7) | | 6g | | |
| Li8 | | 0.000000 | | 0.3205(14) | | 0.500000 | | | 0.66(2) | | 0.087(8) | 6h | | |
|  | | *U*_11_ (Å^2^) | | *U*_22_ (Å^2^) | | *U*_33_ (Å^2^) | | | *U*_12_ (Å^2^) | | *U*_13_ (Å^2^) | | *U*_23_ (Å^2^) |  |
| Y1 | | 0.0109(2) | | 0.0109(2) | | 0.0141(4) | | | 0.00543(11) | | 0 | | 0 |  |
| Y2 | | 0.034(2) | | 0.034(2) | | 0.033(7) | | | 0.0171(10) | | 0 | | 0 |  |
| Y3 | | 0.0160(3) | | 0.0160(3) | | 0.0250(9) | | | 0.00799(13) | | 0 | | 0 |  |
| Cl4 | | 0.0325(7) | | 0.0325(7) | | 0.0311(10) | | | 0.0194(7) | | 0.0035(2) | | -0.0035(2) |  |
| Cl5 | | 0.0264(5) | | 0.0214(9) | | 0.0338(9) | | | 0.0107(4) | | -0.00288(19) | | -0.0058(4) |  |
| Cl6 | | 0.0265(7) | | 0.0148(9) | | 0.0202(10) | | | 0.0074(4) | | 0.00205(14) | | 0.0041(3) |  |
| Li7 | | 0.031(4) | | 0.075(10) | | 0.153(17) | | | 0.038(5) | | 0.032(6) | | 0.064(12) |  |
| Li8 | | 0.071(12) | | 0.054(7) | | 0.14(2) | | | 0.035(6) | | -0.015(17) | | -0.007(8) |  |
|  | |  | |  | |  | | |  | |  | |  |  |

**Table S14.** Refined structure features of different temperature treated Li_3_YCl_6_ (from ball milled sample) and measured Li^+^ ionic conductivity.

|  | Ball milled | 140 °C heat treated and cooled down to RT | 200 °C heat treated and hot pressed | 400 °C annealed and furnace cooled down to RT |
| --- | --- | --- | --- | --- |
| Li^+^ conductivity (mS/cm) at 25 °C | 0.39 | 0.034 | 0.16 | 0.043 |
| Activation energy (eV) (between 25 °C and 80 °C) | 0.52 | 0.51 | 0.68 | 0.56 |
| Y-vacancy ordering features from structure refinements | Limited coherence of honeycomb, zig-zag and linear ordering domains. Large portions of Y-poor domain in both planes | Mixed ordering, scattered honeycomb ordering domains | Mixed ordering, large zig-zag ordering domains | Predominant honeycomb ordering domains |

**References**

(1) Juhas, P.; Davis, T.; Farrow, C. L.; Billinge, S. J. L. PDFgetX3: a rapid and highly automatable program for processing powder diffraction data into total scattering pair distribution functions. *J. Appl. Crystallogr.* **2013**, *46* (2), 560-566.

(2) Neuefeind, J.; Feygenson, M.; Carruth, J.; Hoffmann, R.; Chipley, K. K. The Nanoscale Ordered MAterials Diffractometer NOMAD at the Spallation Neutron Source SNS. *Nucl. Instrum. Methods Phys. Res. B* **2012**, *287*, 68-75. DOI: <https://doi.org/10.1016/j.nimb.2012.05.037>.

(3) Calder, S.; An, K.; Boehler, R.; Dela Cruz, C. R.; Frontzek, M. D.; Guthrie, M.; Haberl, B.; Huq, A.; Kimber, S. A. J.; Liu, J.; et al. A suite-level review of the neutron powder diffraction instruments at Oak Ridge National Laboratory. *Rev. Sci. Instrum.* **2018**, *89* (9), 092701. DOI: 10.1063/1.5033906.

(4) Coelho, A. TOPAS and TOPAS-Academic: an optimization program integrating computer algebra and crystallographic objects written in C++. *J. Appl. Crystallogr.* **2018**, *51* (1), 210-218.

(5) Ikeda, S.; Carpenter, J. M. Wide-energy-range, high-resolution measurements of neutron pulse shapes of polyethylene moderators. *Nucl. Instrum. Methods Phys. Res. A* **1985**, *239* (3), 536-544. DOI: <https://doi.org/10.1016/0168-9002(85)90033-6>.

(6) Liu, J.; Du, Z.; Wang, X.; Tan, S.; Wu, X.; Geng, L.; Song, B.; Chien, P.-H.; Everett, S. M.; Hu, E. Anionic redox induced anomalous structural transition in Ni-rich cathodes. *Energy Environ. Sci.* **2021**, *14* (12), 6441-6454, 10.1039/D1EE02987H. DOI: 10.1039/D1EE02987H.

(7) Young, R. A. *The rietveld method*; International union of crystallography, 1993.

(8) Zhang, Y.; Liu, J.; Tucker, M. G. Lorentz factor for time-of-flight neutron Bragg and total scattering. *Acta Crystallogr. Sect. A* **2023**, *79* (1), 20-24.

(9) Sheldrick, G. Crystal structure refinement with SHELXL. *Acta Crystallographica Section C* **2015**, *71* (1), 3-8.

(10) Giannozzi, P.; Baroni, S.; Bonini, N.; Calandra, M.; Car, R.; Cavazzoni, C.; Ceresoli, D.; Chiarotti, G. L.; Cococcioni, M.; Dabo, I.; et al. QUANTUM ESPRESSO: a modular and open-source software project for quantum simulations of materials. *J. Phys.: Condens. Matter* **2009**, *21* (39), 395502. DOI: 10.1088/0953-8984/21/39/395502.

(11) Giannozzi, P.; Andreussi, O.; Brumme, T.; Bunau, O.; Buongiorno Nardelli, M.; Calandra, M.; Car, R.; Cavazzoni, C.; Ceresoli, D.; Cococcioni, M.; et al. Advanced capabilities for materials modelling with Quantum ESPRESSO. *J. Phys.: Condens. Matter* **2017**, *29* (46), 465901. DOI: 10.1088/1361-648X/aa8f79.

(12) Dal Corso, A. Pseudopotentials periodic table: From H to Pu. *Computational Materials Science* **2014**, *95*, 337-350. DOI: <https://doi.org/10.1016/j.commatsci.2014.07.043>.

(13) Perdew, J. P.; Burke, K.; Ernzerhof, M. Generalized Gradient Approximation Made Simple. *Phys. Rev. Lett.* **1996**, *77* (18), 3865-3868. DOI: 10.1103/PhysRevLett.77.3865.

(14) Ong, S. P.; Richards, W. D.; Jain, A.; Hautier, G.; Kocher, M.; Cholia, S.; Gunter, D.; Chevrier, V. L.; Persson, K. A.; Ceder, G. Python Materials Genomics (pymatgen): A robust, open-source python library for materials analysis. *Computational Materials Science* **2013**, *68*, 314-319. DOI: <https://doi.org/10.1016/j.commatsci.2012.10.028>.

(15) Coelho, A. Whole-profile structure solution from powder diffraction data using simulated annealing. *J. Appl. Crystallogr.* **2000**, *33* (3), 899-908.

(16) Kirkpatrick, S.; Gelatt, C. D.; Vecchi, M. P. Optimization by Simulated Annealing. *Science* **1983**, *220* (4598), 671-680. DOI: 10.1126/science.220.4598.671.

(17) Metropolis, N.; Rosenbluth, A. W.; Rosenbluth, M. N.; Teller, A. H.; Teller, E. Equation of State Calculations by Fast Computing Machines. *The Journal of Chemical Physics* **1953**, *21* (6), 1087-1092. DOI: 10.1063/1.1699114.

(18) McGreevy, R. L. RMC: progress, problems and prospects. *Nucl. Instrum. Methods Phys. Res. A* **1995**, *354* (1), 1-16. DOI: <https://doi.org/10.1016/0168-9002(94)00926-0>.

(19) Voter, A. F. Introduction to the kinetic Monte Carlo method. In *Radiation effects in solids*, Springer, 2007; pp 1-23.
